# RSU-1 interaction with prohibitin-2 links cell–extracellular matrix detachment to downregulation of ERK signaling

**DOI:** 10.1074/jbc.RA120.014413

**Published:** 2020-12-03

**Authors:** Meiling Wang, Jie Liu, Yizeng Tu, Zihan Zhao, Jingjing Qu, Ka Chen, Yonglong Chen, Ying Sun, Hui Zhao, Yi Deng, Chuanyue Wu

**Affiliations:** 1 School of Life Science and Technology, Harbin Institute of Technology, Harbin, China; 2 Department of Biology, Guangdong Provincial Key Laboratory of Cell Microenvironment and Disease Research, and Shenzhen Key Laboratory of Cell Microenvironment, Southern University of Science and Technology, Shenzhen, China; 3 Department of Pathology, University of Pittsburgh School of Medicine, Pittsburgh, Pennsylvania, USA; 4 The Faculty of Health Sciences, The University of Macau, Macau, China; 5 School of Biomedical Sciences, Faculty of Medicine, The Chinese University of Hong Kong, Hong Kong, China

**Keywords:** Ras suppressor 1 (RSU1), extracellular signal–regulated kinase (ERK), prohibitin (PHB), extracellular matrix (ECM), integrin, lipid rafts, CTxB, cholera toxin B subunit, 3D, 3-dimentional, ECM, extracellular matrix, ERK, extracellular signal–regulated kinase, FBS, fetal bovine serum, FLIM, fluorescence lifetime imaging microscopy, FRET, Förster resonance energy transfer, gRNA, guide RNA, ILK, integrin-linked kinase, LRR, leucine-rich repeat, MBP, maltose-binding protein, MEK, mitogen-activated protein kinase kinase, PINCH, particularly interesting new cysteine-histidine-rich protein, TCSPC-FLIM, time-correlated single-photon fluorescence lifetime microscopy

## Abstract

Cell–extracellular matrix (ECM) detachment is known to decrease extracellular signal–regulated kinase (ERK) signaling, an intracellular pathway that is central for control of cell behavior. How cell–ECM detachment is linked to downregulation of ERK signaling, however, is incompletely understood. We show here that focal adhesion protein Ras Suppressor 1 (RSU1) plays a critical role in cell–ECM detachment induced suppression of ERK signaling. We have identified prohibitin 2 (PHB2), a component of membrane lipid rafts, as a novel binding protein of RSU1, and mapped a major RSU1-binding site to PHB2 amino acids 150 to 206 in the C-terminal region of the PHB/SPFH (stomatin/prohibitin/flotillin/HflKC) domain. The PHB2 binding is mediated by multiple sites located in the N-terminal leucine-rich repeat region of RSU1. Depletion of PHB2 suppressed cell–ECM adhesion–induced ERK activation. Furthermore, cell–ECM detachment increased RSU1 association with membrane lipid rafts and interaction with PHB2. Finally, knockout of RSU1 or inhibition of RSU1 interaction with PHB2 by overexpression of the major RSU1-binding PHB2 fragment (amino acids 150–206) effectively suppressed the cell–ECM detachment induced downregulation of ERK signaling. Additionally, expression of venus-tagged wild-type RSU1 restored ERK signaling, while expression of venus-tagged PHB2-binding defective RSU1 mutant in which the N-terminal leucine-rich repeat region is deleted did not. Taken together, Our findings identify a novel RSU1-PHB2 signaling axis that senses cell–ECM detachment and links it to decreased ERK signaling.

It has been well established that cell–extracellular matrix (ECM) adhesion is crucial for regulation of the extracellular signal–regulated kinase (ERK) signaling pathway, which is central for control of cell behavior including cell proliferation, migration, and survival (1, 2, 3, 4, 5, 6, 7, 8, 9). Detachment of cells from the ECM often results in downregulation of ERK activation and consequently alteration of cell behavior (1, 10, 11, 12). Aberrant ERK signaling and anchorage-independent growth are intimately associated with cancer development and progression (13, 14). Thus, elucidation of the molecular mechanisms underlying cell–ECM adhesion–mediated regulation of ERK signaling is important for understanding the pathogenesis of cancer and identifying therapeutic targets for control of cancer progression.

Cell–ECM adhesion triggers recruitment of a selective number of intracellular proteins to focal adhesions, where these proteins or protein complexes act as signaling intermediators regulating cell proliferation, migration, and survival (15, 16, 17, 18, 19, 20, 21). One of the key focal adhesion components is a tertiary protein complex composed of integrin-linked kinase (ILK), particularly interesting new cysteine-histidine-rich protein (PINCH), and parvin (hereinafter referred to as the ILK-PINCH-parvin complex) (19, 22, 23). The ILK-PINCH-parvin complex binds to other adaptor proteins as well as actin cytoskeleton, thereby regulating various signaling pathways critical for cell-cycle progression, cell migration, and survival (22, 23, 24, 25, 26). The formation of the ILK-PINCH-parvin complex is mediated by the interactions of the ILK amino- and carboxyl-terminal domains to PINCH and parvin, respectively (22). PINCH also interacts with RSU1, a leucine-rich repeat (LRR)–containing protein that was originally identified and named after its ability to suppress *v*-Ki-Ras–induced oncogenic transformation (27, 28, 29). The interaction with PINCH recruits RSU1 to focal adhesions (19, 30, 31, 32). Functionally, both clinical evidence and experimental studies in model organisms and cells have indicated that ILK, PINCH, and parvin play important roles in promoting tumor development and progression (22, 23, 25, 33, 34, 35). While RSU1 has been found to play a positive role in cancer cell migration and invasion (36), substantial evidence suggests that RSU1 may also function as a tumor suppressor. For example, loss or reduced expression or mutations of *RSU1* was often found in human cancers (*e.g.*, hepatocellular carcinoma and gliomas) (37, 38, 39). Furthermore, overexpression of RSU1 significantly reduced human breast cancer and glioblastoma cell growth and tumorigenic potential (39, 40). How RSU1 suppresses oncogenic transformation and tumor growth, however, is not well understood.

Lipid rafts are membrane microdomains found in eukaryotic cells, which are enriched in cholesterol, sphingolipid, and many lipid-linked proteins including caveolin (41, 42). It has been shown that membrane lipid rafts are discrete platforms for anchoring important signaling molecules such as ERK and its upstream kinases such as mitogen-activated protein kinase kinase (MEK) to transduce signals critical for cell proliferation, growth, and migration (43, 44, 45, 46). Of note, inhibition of caveolin-mediated internalization of lipid rafts effectively prevented cell–ECM detachment induced downregulation of ERK signaling (47, 48). PHB2 is a component of the ubiquitously expressed PHB complex that is present in various subcellular locations including membrane lipid rafts (49, 50). Structurally, PHB2 is composed of an amino-terminal hydrophobic sequence that facilities its localization to the membrane, a large PHB/SPFH domain known to have affinity for binding to lipid rafts, and a carboxyl-terminal coiled-coil domain required for the assembly of the PHB complex (49). Previous studies have shown that the PHB complex is essential for Ras-induced ERK activation (51, 52, 53). Furthermore, rocaglamides, natural compounds that potently inhibit proliferation of various cancer cells, have been shown to directly bind the PHB complex and thereby inhibit ERK signaling (54, 55).

In the current study, we show that RSU1 plays a critical role in cell–ECM detachment induced downregulation of ERK signaling. We have identified PHB2 as a novel binding protein of RSU1 and mapped a major RSU1-binding site to PHB2 amino acids 150 to 206 in the C-terminal region of the PHB/SPFH domain. In addition, we have found that multiple sites in the N-terminal LRR region of RSU-1 mediate the PHB2 binding. Functionally, depletion of PHB2 suppressed cell–ECM adhesion–induced ERK activation. Furthermore, cell–ECM detachment promoted RSU1 association with membrane lipid rafts and interaction with PHB2. Finally, knockout (KO) of RSU1 or inhibition of RSU1 interaction with PHB2 by overexpression of the RSU1-binding PHB2 fragment (amino acids 150–206) effectively suppressed the cell–ECM detachment–induced downregulation of ERK signaling. Expression of venus-tagged wild-type RSU1, but not that of venus-tagged PHB2-binding defective RSU1 mutant in which the N-terminal LRR region is deleted, restored cell–ECM detachment–induced downregulation of ERK signaling. Our results identify a novel RSU1-PHB2 signaling axis that senses cell–ECM detachment and links it to downregulation of ERK signaling.

## Results

### RSU1 is critical for cell–ECM detachment–induced downregulation of the ERK signaling pathway

**Figure 1:**
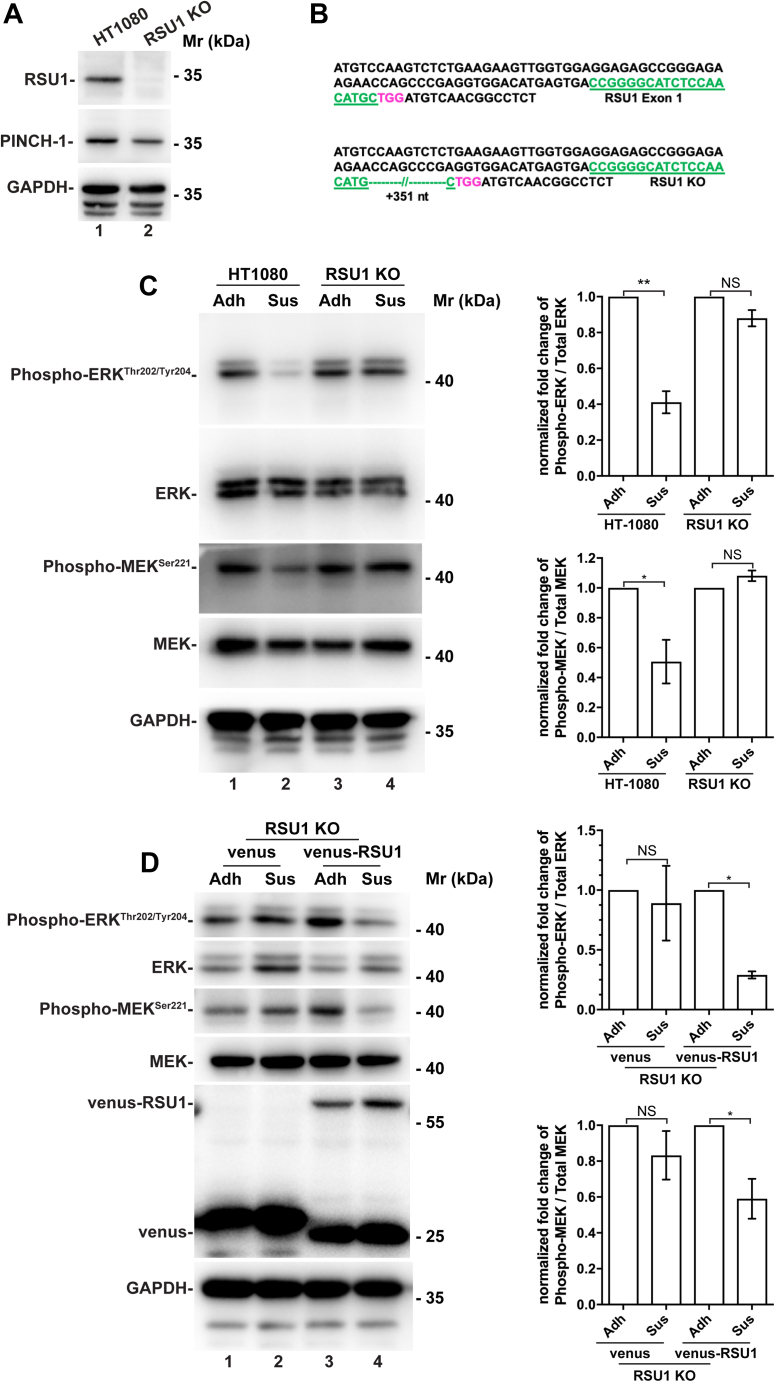
**RSU1 deficiency impairs cell–ECM detachment–induced downregulation of MEK and ERK signaling.***A*, verification of RSU1 deficiency in RSU1 KO cells by Western blotting. HT1080 (lane 1) and RSU1 KO (lane 2) cells were analyzed by Western blotting with anti-RSU1, anti-PINCH-1, and GAPDH antibodies as described in Experimental procedures. *B*, verification of RSU1 disruption in RSU1 KO cells by DNA sequencing. DNA sequencing revealed that a 351-nucleotide (nt) insertion was detected at the expected cleavage site (shown in *red*) in RSU1 KO cells resulting in frameshift and early termination of RSU1. The protospacer adjacent motif (PAM) is shown in *red*, and the gRNA-targeting sites are shown in *green*. *C*, RSU1-deficient cells failed to downregulate MEK and ERK signaling in response to cell detachment from the ECM. Wild-type and RSU1 KO HT1080 cells were either allowed to adhere to fibronectin (10 μg/ml) (Adh) or maintained in suspension in HEMA-coated cell culture dishes (Sus) for 5 h. The levels of total MEK and ERK and phosphorylated MEK^Ser 221^ and ERK^Thr202/Tyr204^ were determined by Western blotting. The densiometric ratios of phosphorylated MEK^Ser 221^ to the total MEK and that of phosphorylated ERK^Thr202/Tyr204^ to the total ERK were analyzed as described in Experimental procedures. In each data set, data were normalized to those observed in adherent cells. Differences between the attached and suspended cells were examined for statistical significance as described in Experimental procedures. n = 4 experiments, ∗*p* < 0.05. *D*, re-expression of RSU1 in RSU1-deficient cells restored downregulation of MEK–ERK signaling in response to loss of cell–ECM adhesion. RSU1 KO cells stably expressing venus-tagged RSU1 or venus alone were cultured in adhesion and suspension conditions, respectively, and the levels of total MEK and ERK and phosphorylated MEKSer 221 and ERKThr202/Tyr204 were assessed as described in (*C*). n = 4 experiments, ∗*p* < 0.05. ECM, extracellular matrix; ERK, extracellular signal–regulated kinase; HEMA, hydroxyethyl methacrylate; KO, knockout; MEK, mitogen-activated protein kinase kinase; NS, not significant.

To facilitate the studies on RSU1, we knocked out *RSU1* from human HT1080 fibrosarcoma cells (hereinafter referred to as RSU1 KO cells) using the CRISPR/Cas9 techniques with the gRNA directing to exon 1 of *RSU1*. DNA sequencing revealed insertion mutations at *RSU1* loci (Fig. 1*B*), and the disruption of *RSU1* was further confirmed by Western blotting with anti-RSU1 antibody (Fig. 1*A*). Next, we tested the role of RSU1 in cell–ECM adhesion–induced regulation of MEK and ERK signaling. To do this, we allowed wild-type HT1080 cells and RSU1 KO cells to get adhered to fibronectin-coated surface or maintained them in suspension for the same period of time. As expected, loss of cell–ECM adhesion significantly reduced the levels of activating phosphorylation of MEK and ERK in wild-type HT1080 cells (Fig. 1*C*, compare lanes 1 and 2). The densiometric ratios of phosphorylated MEK to total MEK and that of phosphorylated ERK to total ERK were reduced to 51% (*p* < 0.05) and 41% (*p* < 0.01), respectively, in suspended HT1080 cells compared with those in HT1080 cells adhered to fibronectin (Fig. 1*C*). By marked contrast, the cell–ECM detachment–induced suppression of MEK and ERK activation was abolished in the absence of RSU1 (Fig. 1*C*, compare lanes 3 and 4), suggesting that RSU1 is essential for cell–ECM detachment–induced suppression of MEK and ERK activation. To confirm these results, we re-expressed RSU1 or the empty vector as a control in the RSU1 KO cells. Cell–ECM detachment–induced suppression of MEK and ERK activation was restored in the cells re-expressing RSU1 (Fig. 1*D*, compare lanes 3 and 4) but not in the control cells (Fig. 1*D*, compare lanes 1 and 2), confirming an essential role of RSU1 in this process. We have also tested the role of RSU1 in cell–ECM adhesion–induced regulation of MEK and ERK signaling in other cell types (*e.g.*, human MDA-MB-231 breast cancer cells), in which RSU1 was depleted using the same CRISPR/Cas9 strategy. The results showed that similar to what we found in HT1080 cells, loss of RSU1 in MDA-MB231 cells impaired the cell–ECM detachment–induced suppression of MEK and ERK activation (Fig. S1). Again, re-expression of RSU1 in the RSU1 KO MD231 cells restored the cell–ECM detachment–induced suppression of MEK and ERK activation (Fig. S1). These results suggest that RSU1 is critically involved in the suppression of MEK and ERK activation in response to loss of cell–ECM adhesion.

### Identification of PHB2 as an RSU1-binding protein

**Figure 2:**
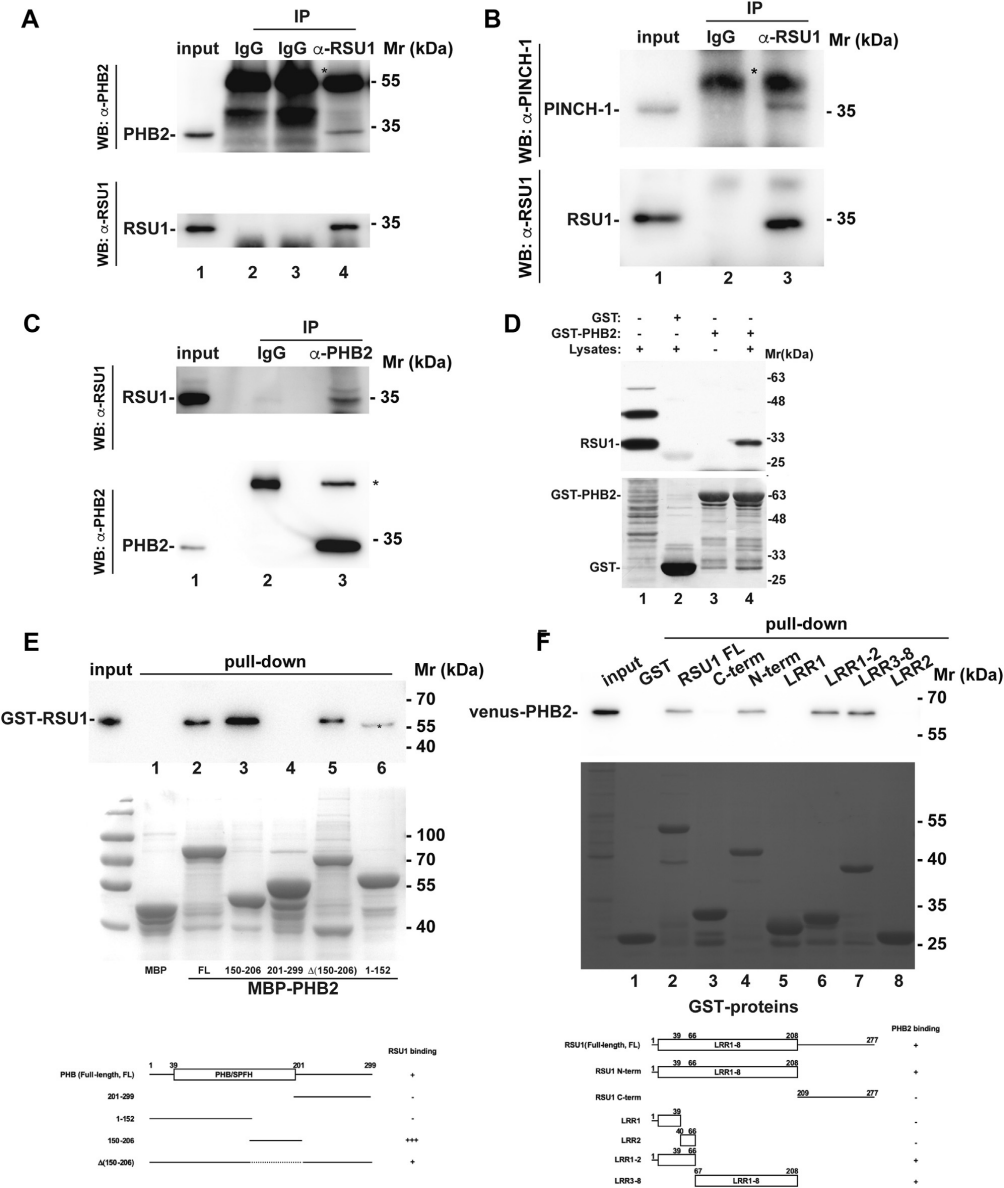
**RSU1 interacts with PHB2.***A*–*B*, coimmunoprecipitation of PHB2 (*A*) and PINCH-1 (*B*) with RSU1. HT1080 cells were harvested and lysed with octyl glucoside (OG)–containing lysis buffer. Endogenous RSU1 was immunoprecipitated with anti-RSU1 antibody and mouse IgG as a negative control, and the immunoprecipitants were analyzed by Western blotting with anti-RSU1 (*A* and *B*), anti-PHB2 (*A*), and anti-PINCH-1 (*B*) antibodies. The heavy chain of IgG is marked by *asterisk*. *C*, coimmunoprecipitation of RSU1 with PHB2. HT1080 cells were harvested and lysed with OG-containing lysis buffer. Endogenous PHB2 was immunoprecipitated with anti-PHB2 antibody and mouse IgG as a negative control, and the immunoprecipitants were analyzed by Western blotting with anti-RSU1 and anti-PHB2 antibodies. The heavy chain of IgG is marked by *asterisk*. *D*, Pull down of RSU1 by GST-PHB2. GST pull-down assay was performed as described in Experimental procedures. The samples (as indicated) were analyzed by Western blotting with anti-RSU1 antibody (*left*) and Coomassie blue staining (*right*), respectively. GST was served as a negative control. *E*, recombinant MBP-fusion proteins containing full-length (FL) PHB2, PHB2 N-terminal region (aa 1–152), middle region (aa 150–206), or C-terminal region (aa 201–299) or PHB2 mutant in which aa 150 to 206 are deleted (Δ150–206) were expressed and purified, and their interactions with GST-tagged RSU1 were analyzed using a pull-down assay as described in the Experimental procedures. MBP was used as a negative control. The samples were analyzed by Western blotting with anti-RSU1 antibody (*top*) or Coomassie blue staining (*middle*). In the Western blot, a protein band with molecular mass identical to MBP-PHB2 C-terminal region (aa 201–299), which is smaller than that of the GST-RSU1 band, was indicated with *asterisk* (lane 6). This band was likely caused by a cross-reactivity of the anti-RSU1 antibody used for the Western blotting. Bottom panel, a schematic drawing summarizing the RSU1-binding activities of the FL and deletion mutants of PHB2 used in the pull-down assay. The PHB/SPFH domain (amino acid residues 39–201) known to have affinity for binding to lipid rafts is marked. *F*, recombinant GST-fusion proteins containing different regions of RSU1 (as indicated) were used to pull down venus-PHB2 expressed in HT1080 cells. The samples were analyzed by Western blotting with anti-PHB2 antibody (*top*) and Coomassie blue staining (*middle*). Bottom panel, a schematic drawing summarizing the PHB2-binding activities of the FL and deletion mutants of RSU1 used in the pull-down assay. MBP, maltose-binding protein; PINCH, particularly interesting new cysteine-histidine-rich protein; PHB2, prohibitin 2; SPFH, stomatin/prohibitin/flotillin/HflKC.

We next investigated the mechanism by which RSU1 functions to suppress MEK and ERK signaling in response to cell–ECM detachment. Because RSU1 does not possess intrinsic catalytic activity, we postulated that it might function in this process through interacting with a key regulator of the MEK–ERK signaling pathway. To begin to test this, we performed yeast two-hybrid cDNA library screening using RSU1 as bait and identified PHB2, a component of the PHB complex that is critical for regulation of the MEK–ERK signaling pathway (51), as a novel binding partner of RSU1 (see Experimental procedures). To validate that RSU1 forms a complex with PHB2 in cells, we carried out coimmunoprecipitation experiments using anti-RSU1 and anti-PHB2 antibodies, respectively. The results showed that PHB2 (Fig. 2*A*, lane 4), like PINCH-1 (Fig. 2*B*, lane 3), was coimmunoprecipitated with RSU1. No PHB2 or PINCH-1 was detected in the control IgG precipitants (Fig. 2*A*, lanes 2 and 3; Fig. 2*B*, lane 2). Reciprocally, RSU1 was detected in anti-PHB2 (Fig. 2*C*, lane 3) but not control IgG immunoprecipitants (Fig. 2*C*, lane 2). We have also analyzed the interaction between RSU1 and PHB2 using GST-fusion protein pull-down assay. Recombinant GST–PHB2 fusion protein was expressed and tested whether it could pull down RSU1 from mammalian cells (Fig. 2*D*). Consistent with the coimmunoprecipitation results, RSU1 was readily pulled down by GST–PHB2 (Fig. 2*D*, lane 4) but not by GST lacking PHB2 sequence (Fig. 2*D*, lane 2). Collectively, these results confirm that RSU1 and PHB2 form a complex in mammalian cells.

To test whether RSU1 directly interacts with PHB2, we expressed recombinant GST-tagged RSU1 and maltose-binding protein (MBP)–tagged PHB2 in *Escherichia coli*, respectively, and analyzed the ability of purified GST-RSU1 to interact with MBP–PHB2 in pull-down assays. The results showed that GST-RSU1 was readily pulled down by MBP–PHB2 (Fig. 2*E*, lane 3) but not by MBP (Fig. 2*E*, lane 2), suggesting that RSU1 binds to PHB2 directly. Next, we generated MBP-tagged PHB2 fragments containing the N-terminal region (amino acids 1–152), the middle region (amino acids 150–206), or the C-terminal region (amino acids 201–299) and tested their RSU1-binding activity using the pull-down assays. The results showed that GST-RSU1 was pulled down by MBP-tagged PHB2 fragment containing the middle region (amino acids 150–206) (Fig. 2*E*, lane 3) but not by that containing the C-terminal region (amino acids 201–299) (Fig. 2*E*, lane 4) or the N-terminal region (amino acids 1–152) (Fig. 2*E*, lane 6), suggesting that PHB2 middle region (amino acids 150–206) contains a major RSU1-binding site. However, GST-RSU1 was pulled down by MBP-tagged PHB2 mutant in which amino acids 150 to 206 were deleted (Δ(150–206)) (Fig. 2*E*, lane 5), albeit in a smaller amount compared with that of GST-RSU1 pulled down by the PHB2 middle region (amino acids 150–206) (Fig. 2*E*, compare lane 3 with lane 5), suggesting that deletion of amino acids 150 to 206 does not completely eliminate RSU1 binding.

Next, we sought to identify the RSU1 region that mediates PHB2 binding. To do this, we generated GST-tagged proteins containing various RSU1 regions (Fig. 2*F*) and tested their PHB2-binding activity in a pull-down assay. The results showed that GST-tagged RSU1 N-terminal LRR region (amino acids 1–208) (Fig. 2*F*, lane 4), but not GST-tagged RSU1 C-terminal region (amino acids 209–277) (Fig. 2*F*, lane 3), pulled down venus-tagged PHB2. In control experiments, venus-tagged PHB2 was pulled down by GST-RSU1 (Fig. 2*F*, lane 2) but not by GST (Fig. 2*F*, lane 1), confirming the specificity of the pull-down assay. Venus-tagged PHB2 was also pulled down by GST-tagged LRR1-LRR2 (amino acids 1–66) (Fig. 2*F*, lane 6) or LRR3-LRR8 (amino acids 67–208) (Fig. 2*F*, lane 7) but not LRR1 (amino acids 1–43) (Fig. 2*F*, lane 5) or LRR2 (amino acids 44–66) (Fig. 2*F*, lane 8). Collectively, these results suggest that the PHB2 binding is mediated by multiple LRRs located in the N-terminal region (amino acids 1–208) of RSU1.

### PHB2 is critical for cell–ECM adhesion–induced MEK and ERK activation

**Figure 3:**
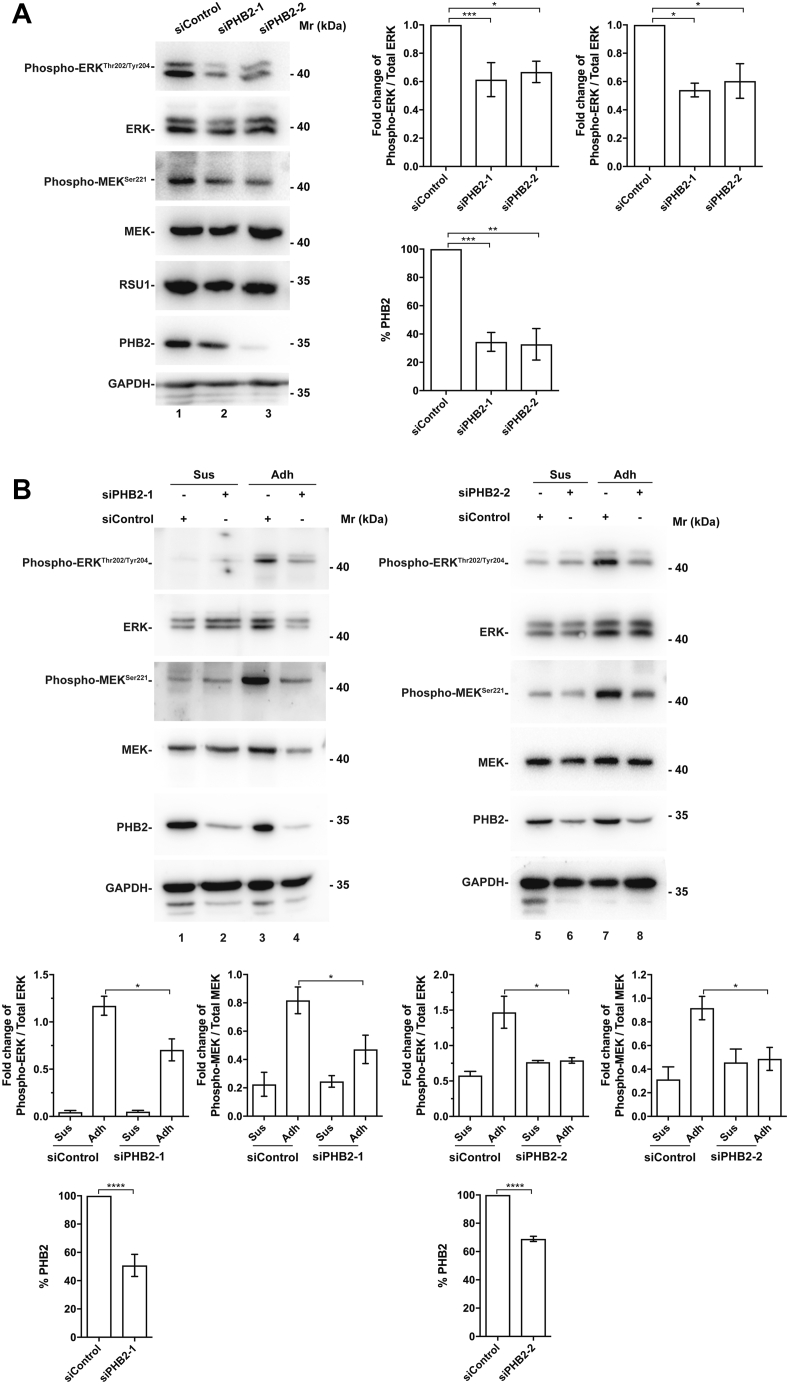
***PHB2* silencing impairs cell–ECM adhesion–induced MEK and ERK activation.***A*, silencing *PHB2* with siRNA results in downregulation of MEK and ERK activation under basal condition. HT1080 cells transfected with control siRNA (siControl) or two different PHB2 siRNAs (siPHB2-1 and siPHB2-2) for 48 h were harvested and examined for the levels of total MEK and ERK and phosphorylated MEK^Ser 221^ and ERK^Thr202/Tyr204^ by Western blotting. The densiometric ratio of PHB2 to GAPDH was used to indicate the knockdown efficiency of PHB2. The densiometric ratio of phosphorylated MEK^Ser 221^ to the total MEK and that of phosphorylated ERK^Thr202/Tyr204^ to the total ERK were analyzed as described in Experimental procedures. In each data set, data were normalized to those observed in cells transfected with siControl. Differences between the adherent and suspended cells were examined for statistical significance as described in Experimental procedures. n = 5 experiments, ∗*p* < 0.05, ∗∗*p* < 0.005, ∗∗∗*p* < 0.001. *B*, *PHB2* silencing impairs cell–ECM adhesion–induced MEK and ERK activation. HT1080 cells transfected with control siRNA (siControl) or two different PHB2 siRNAs (siPHB2-1 and siPHB2-2) for 48 h were trypsinized and then were either maintained in suspension in HEMA-coated cell culture dishes (Sus) or allowed to adhere to fibronectin (10 μg/ml) (Adh) for 5 h. The cells were analyzed by Western blotting with antibodies as indicated. The densiometric ratio of phosphorylated MEK^Ser 221^ to the total MEK and that of phosphorylated ERK^Thr202/Tyr204^ to the total ERK were analyzed as described in Experimental procedures. n = 3 experiments, ∗*p* < 0.05. ∗∗∗∗*p* < 0.0001. ECM, extracellular matrix; ERK, extracellular signal–regulated kinase; HEMA, hydroxyethyl methacrylate.

We next tested whether PHB2 is involved in the regulation of cell–ECM adhesion–induced MEK and ERK activation. To do this, we knocked down PHB2 from HT1080 cells by siRNA (Fig. 3*A*). Consistent with previous studies (51, 53, 55), knockdown of PHB2 in HT1080 cells with two different PHB2 siRNAs significantly reduced the activating phosphorylation of MEK and ERK under normal culture condition (Fig. 3*A*). To test the effect on cell–ECM adhesion–induced MEK and ERK activation, both PHB2 knockdown and the control cells that express a normal level of PHB2 were either maintained in suspension or allowed to adhere to fibronectin for 5 h. As expected, the levels of the activating phosphorylation of MEK and ERK in control cells were markedly increased in response to cell adhesion to fibronectin (Fig. 3*B*, compare lane 1 with lane 3, and lane 5 with lane 7; lower panels). The cell–ECM adhesion–induced increase of the activating phosphorylation of MEK and ERK was significantly reduced in response to knockdown of PHB2 (Fig. 3*B*, compare lane 4 with lane 3, and lane 8 with lane 7; lower panels). These results suggest that PHB2 is critically involved in the cell–ECM adhesion–induced MEK and ERK activation.

### Cell–ECM detachment promotes RSU1 association with membrane lipid rafts

**Figure 4:**
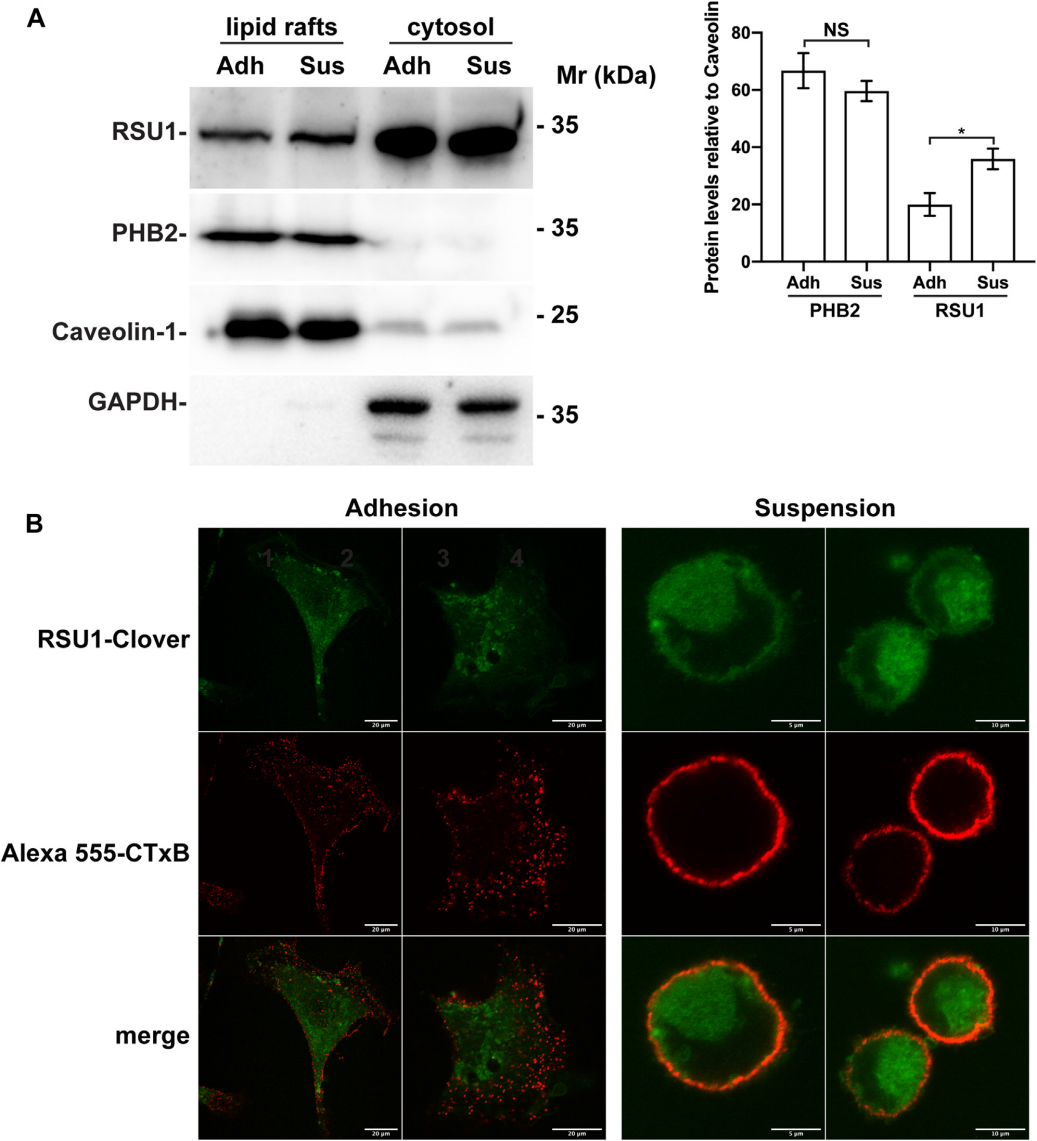
**Cell–ECM detachment promotes RSU1 association with membrane lipid rafts.***A*, distribution of caveolin, PHB2, and RSU1 in lipid rafts and cytosol prepared from HT1080 cells that were either maintained in suspension or allowed to adhere to fibronectin (10 μg/ml). Lipid rafts were isolated from the total membrane fraction as described in Experimental procedures. The blots were probed with antibodies against caveolin-1, GAPDH, PHB2, and RSU1. Noted that the amount of caveolin-1 associated with lipid rafts was similar in both adherent and suspended cells. The densiometric ratio of PHB2 or RSU1 protein levels relative to caveolin-1 was analyzed as described in Experimental procedures. Noted that while PHB2 present in lipid rafts remained essentially the same in suspended and attached cells, the amount of RSU1 detected in lipid rafts was significantly increased in suspended cells compared with that in attached cells. n = 5 experiments, ∗*p* < 0.05, *B*, Colocalization of RSU1-Clover with lipid rafts. The RSU1-Clover HT1080 cells in which the DNA sequence encoding Clover was inserted immediately to the 3ʹof *RSU1* loci were maintained in suspension or allowed to adhere to fibronectin (10 μg/ml) for 5 h before incubation with cholera toxin B subunit (CTxB), a marker for lipid rafts, as described in Experimental procedures. Cells were analyzed by confocal microscopy, and representative images were shown. Note that an increased fraction of RSU1-Clover was colocalized with Alexa555-CTxB in suspended cells. Bars = 20 μm, 5 μm, or 10 μm as indicated in the figure. NS, not significant.

Previous studies suggest that membrane lipid rafts play an important role in cell–ECM adhesion–mediated regulation of ERK activation (47). Furthermore, a fraction of PHB2 is known to be present in membrane lipid rafts (49, 50). Thus, we tested whether RSU1 is physically associated with membrane lipid rafts. To do this, we isolated membrane lipid rafts and cytosol fractions from the cells that were either adhered to fibronectin or maintained in suspension and analyzed RSU1 by Western blotting (Fig. 4*A*). As expected, GAPDH, a cytosolic protein, was detected in the cytosol but not in membrane lipid raft fractions, whereas caveolin, a component of membrane lipid rafts, was detected primarily in the membrane lipid raft fractions (Fig. 4*A*). Although the majority of RSU1 was detected in the cytosol fractions (Fig. 4*A*, lanes 3 and 4), fractions of RSU1 were detected in lipid rafts in which abundant caveolin-1 and PHB2 were present (Fig. 4*A*, lanes 1 and 2). Of note, the level of RSU1 in lipid rafts was significantly (*p* < 0.05%) increased in response to cell–ECM detachment (Fig. 4*A*, right panel). By contrast, cell–ECM detachment did not significantly change the level of PHB2 in lipid rafts (Fig. 4*A*, right panel). These results suggest that the level of RSU1, but not that of PHB2, in lipid rafts is increased in response to loss of cell–ECM adhesion.

To further analyze this, we engineered cells in which Clover, a green fluorescence tag, was inserted immediately to the 3ʹof *RSU1* loci to allow tracing subcellular localization of endogenous RSU1 (hereinafter RSU1-Clover). As expected, in cells that were adhered to fibronectin, abundant RSU1-Clover was detected in focal adhesions (Fig. S2). To detect lipid rafts by florescence confocal microscopy, we stained the cells with cholera toxin B subunit (CTxB), a marker for lipid rafts. Consistent with the biochemical analyses of lipid rafts (Fig. 4*A*, lanes 1 and 3), confocal microscopic analyses of RSU1-Clover and CTxB-positive lipid rafts showed that RSU1-Clover was largely not colocalized with the lipid rafts in adhered cells (Fig. 4*B*, left panels). However, significantly more RSU1-Clover was found to colocalize with CTxB-positive membrane lipid rafts in response to loss of cell–ECM adhesion (Fig. 4*B*, right panels). These results are highly consistent with those of the biochemical assays (Fig. 4*A*), and collectively, they suggest that loss of cell–ECM adhesion promotes RSU1 association with membrane lipid rafts.

### Cell–ECM detachment promotes RSU1 interaction with PHB2

**Figure 5:**
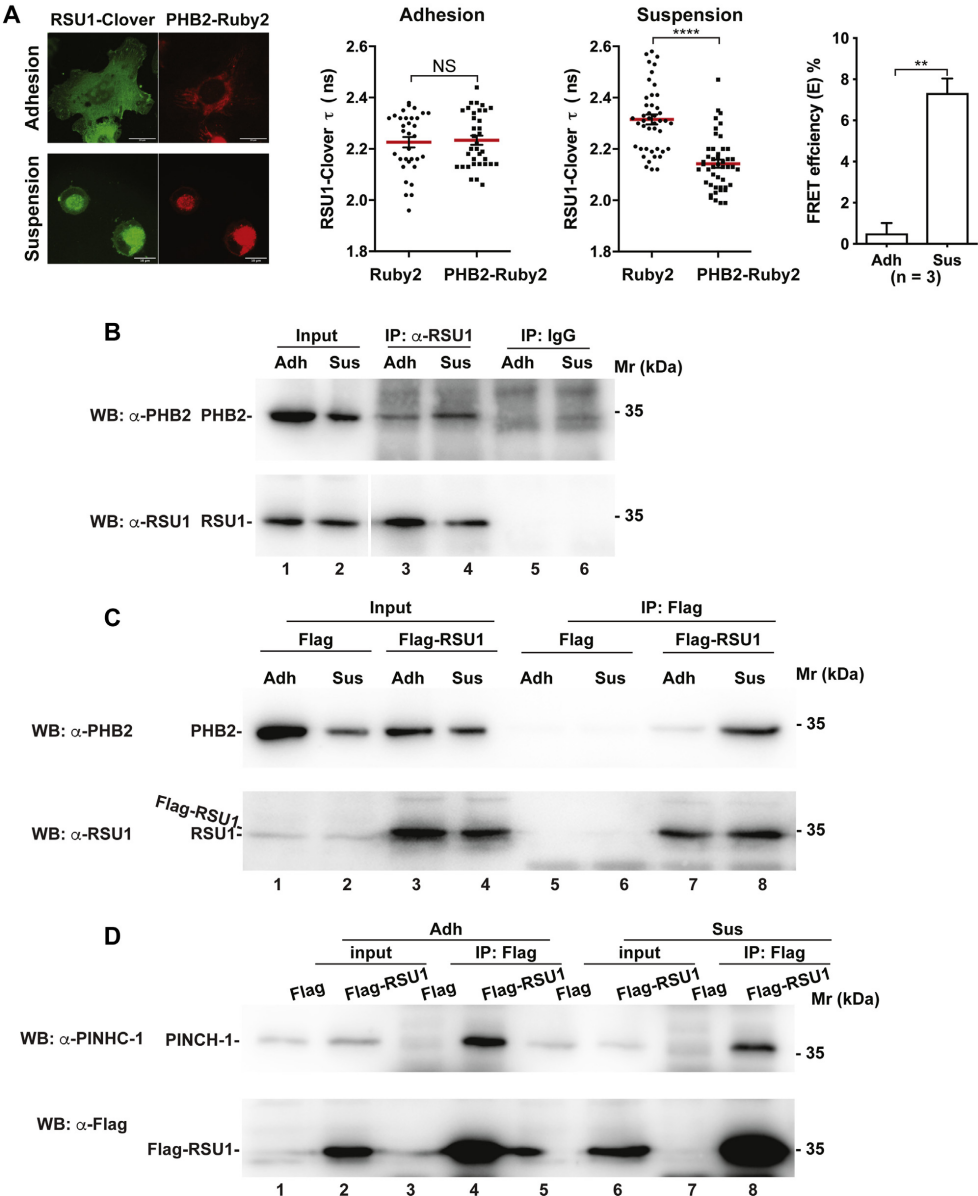
**Cell–ECM detachment promotes RSU1 interaction with PHB2.***A*, FRET analysis of RSU1 interaction with PHB2. HT1080 cells were cotransfected with mClover-N1 vector carrying RSU1 (RSU1-Clover) and Ruby2-N1 vector carrying PHB2 (PHB2-Ruby2) or Ruby2-N1 vector alone as a negative control. The transfected cells were allowed to adhere to fibronectin (10 μg/ml) or maintained in suspension for 5 h before fixation with 4% PFA as described in Experimental procedures (*A*). Bars = 20 μm or 10 μm as indicated in the figure. The fluorescence lifetime (τ) of RSU1-Clover was measured as described in Experimental procedures. The mean ± SEM of τ is plotted. In adherent cells, τ_RSU1-Clover/Ruby2_ = 2.226 ± 0.02 ns (n = 33), τ_RSU1-Clover/PHB2-Ruby2_ = 2.234 ± 0.018 ns (n = 35). In suspended cells, τ_RSU1-Clover/Ruby2_ = 2.315 ± 0.019 ns (n = 44), τ_RSU1-Clover/PHB2-Ruby2_ = 2.143 ± 0.015 ns (n = 46). Noted that in suspended cells, τ_RSU1-Clover_ in the presence of PHB2-Ruby2 was significantly reduced compared with that in the presence of Ruby2 control. ∗∗∗∗*p* < 0.0001, FRET efficiency (E) was calculated as described in Experimental procedures. n = 3, ∗∗*p* < 0.005. *B*, coimmunoprecipitation of PHB2 with endogenous RSU1 in attached and suspended HT1080 cells, respectively. HT1080 cells were trypsinized and seeded on fibronectin (10 μg/ml) or maintained in suspension for 5 h. The cells were then harvested and lysed with octyl glucoside (OG)–containing lysis buffer for immunoprecipitation experiments with anti-RSU1 antibodies as described in Experimental procedures. The anti-RSU1 IgG and control IgG immunoprecipitants were analyzed by Western blotting. Note that the amount of PHB2 coprecipitated with RSU1 in suspended cells was more than that in attached cells. *C* and *D*, coimmunoprecipitation of PHB2 (*C*) or PINCH-1 (*D*) with Flag-RSU1 in attached and suspended HT1080 cells that express Flag-RSU1. HT1080 cells were transfected with Flag-RSU1 or control Flag vector and analyzed by immunoprecipitation with anti-Flag antibodies. The immunoprecipitants were analyzed by Western blotting with antibodies as indicated. Note that the amount of PHB2 (compare lanes 7 and 8 in panel *C*) but not that of PINCH-1 (compare lanes 4 and 8 in panel *D*) coprecipitated with Flag-RSU1 was increased in response to cell–ECM detachment. ECM, extracellular matrix; FRET, Förster resonance energy transfer; PFA, polyformaldehyde; PINCH, particularly interesting new cysteine-histidine-rich protein.

We next sought to test whether cell–ECM adhesion regulates RSU1 interaction with PHB2. We first tested this using Förster resonance energy transfer (FRET) and fluorescence lifetime imaging microscopy (FLIM). HT1080 cells were cotransfected with expression vectors encoding RSU1-Clover and PHB2-Ruby2 or RSU1-Clover and Ruby2 as a negative control and then were either adhered to fibronectin or maintained in suspension. The fluorescence lifetime (τ) of RSU1-Clover was measured to assess FRET efficiency (E) between RSU1-Clover and PHB2-Ruby2 or Ruby2. In cells that were adhered to fibronectin, the fluorescence lifetime of RSU1-Clover in the presence of PHB2-Ruby2 (τ = 2.234 ± 0.018 ns, n = 35) was similar to that in the presence of Ruby alone (τ = 2.226 ± 0.020 ns, n = 33) with barely detectable FRET E (Fig. 5*A*). In contrast, in suspended cells, the fluorescence lifetime of RSU1-Clover in the presence of PHB2-Ruby2 (τ = 2.143 ± 0.015 ns, n = 46) was significantly reduced compared with that in the presence of Ruby2 alone (τ = 2.315 ± 0.019 ns, n = 44) (*p* < 0.0001) with a FRET E of approximately 7.3 % (*p* < 0.005) (Fig. 5*A*). Similar results were obtained when the fluorescence lifetime of PHB2-Clover was analyzed in the presence of RSU1-Ruby2 in suspended HT1080 cells (Fig. S3). These results indicate that loss of cell–ECM adhesion significantly increases the RSU1–PHB2 interaction.

To further test this, we analyzed the interaction between RSU1 and PHB2 in cells that were adhered to the ECM or maintained in suspension by coimmunoprecipitation. The RSU1 immunoprecipitants were analyzed by Western blotting with antibodies for RSU1 and PHB2, respectively. The results showed that the amount of PHB2 coimmunoprecipitated with RSU1 was increased in suspended cells compared with that in adherent cells (Fig. 5*B*, compare lanes 3 and 4). As an additional test, we transfected HT1080 cells with expression vectors encoding FLAG-RSU1 or FLAG only as a control, allowed them to adhere to fibronectin or maintained them in suspension, and analyzed the interaction between PHB2 and FLAG-RSU1 by coimmunoprecipitation with anti-FLAG antibodies (Fig. 5*C*). Again, significantly more PHB2 was coimmunoprecipitated with FLAG-RSU1 in the sample derived from the suspended cells than that derived from the cells adhered to fibronectin (Fig. 5*C*, compare lane 8 with lane 7). These results are highly consistent with those of the FRET experiments, and collectively, they demonstrate that loss of cell–ECM adhesion promotes RSU1 interaction with PHB2. In parallel experiments, the amount of PINCH-1 coimmunoprecipitated with FLAG-RSU1 was not increased in response to cell–ECM detachment (Fig. 5*D*, compare lane 8 with lane 4), suggesting that cell–ECM detachment selectively enhances the interaction of RSU1 with PHB2.

### Overexpression of GFP-PHB2 fragment (150–206) reduces the RSU1–PHB2 interaction and suppresses cell–ECM detachment–induced downregulation of ERK signaling

**Figure 6:**
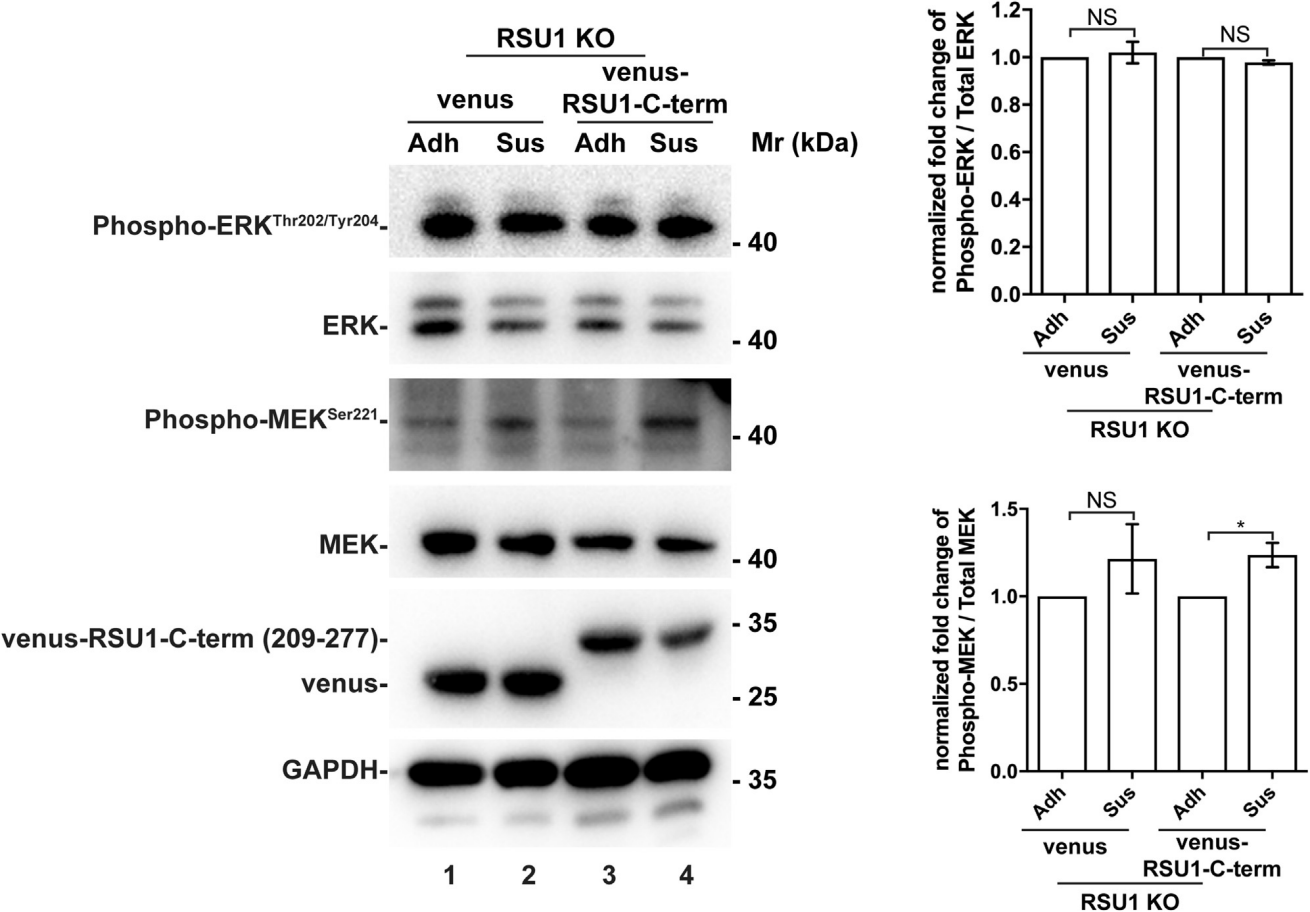
**PHB2-binding defective RSU1 deletion mutant is unable to suppress MEK/ERK activation in response to loss of cell–ECM adhesion**. RSU1 KO cells re-expressing venus-tagged PHB2-binding defective RSU1 mutant (209–277) or venus were allowed to adhere to fibronectin (10 μg/ml) (Adh) or maintained in suspension in HEMA-coated cell culture dishes (Sus), and the levels of total MEK and ERK and phosphorylated MEK^Ser 221^ and ERK^Thr202/Tyr204^ were analyzed by Western Blotting and quantified as described in Figure 1*D*. n = 3 experiments. ECM, extracellular matrix; ERK, extracellular signal–regulated kinase; KO, knockout; HEMA, hydroxyethyl methacrylate; NS, not significant.

**Figure 7:**
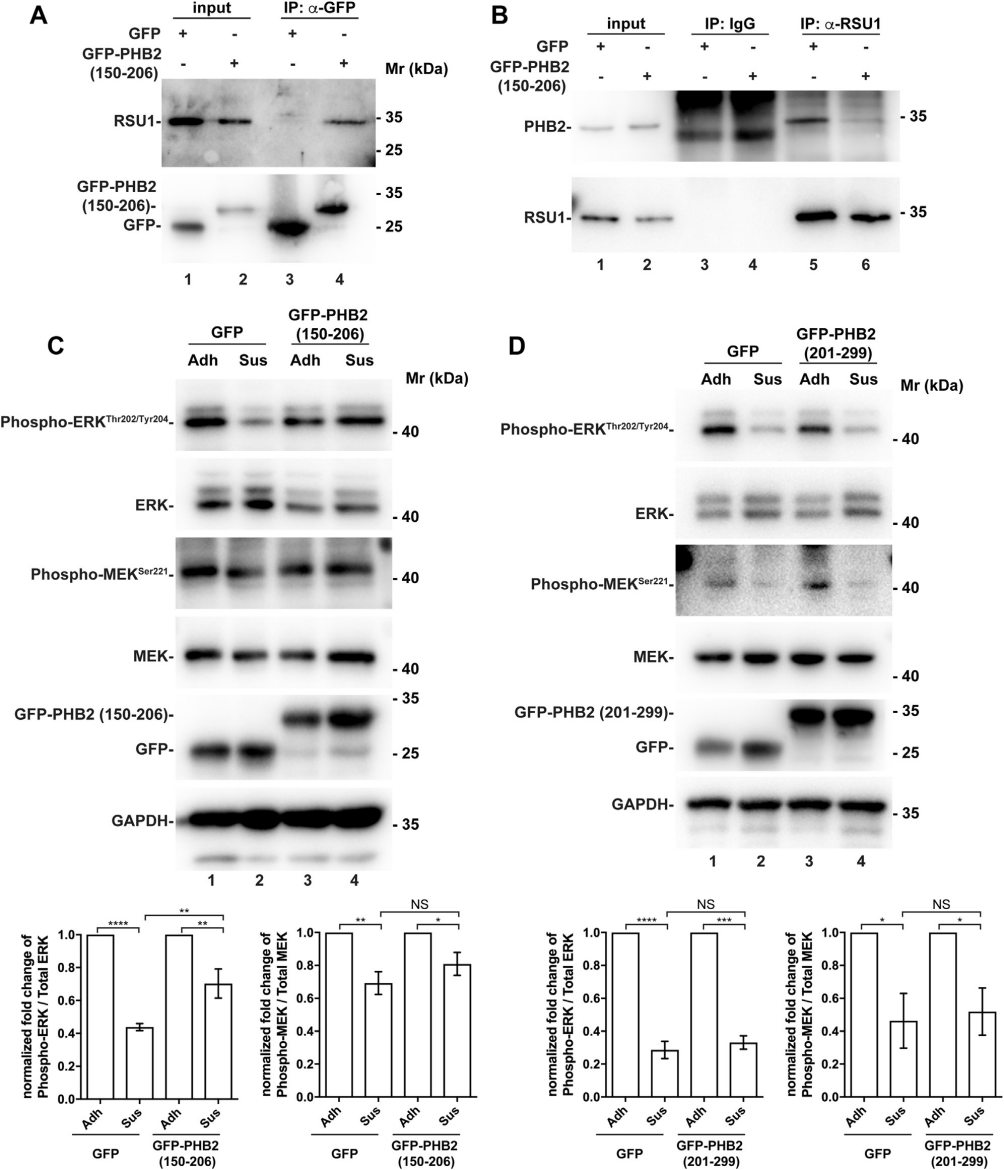
**Overexpression of GFP-PHB2 fragment (150–206) reduces the RSU1–PHB2 interaction and suppresses cell–ECM detachment–induced downregulation of ERK signaling.***A*, coimmunoprecipitation of RSU1 with PHB2 fragment (150–206). HT1080 cells were transfected with vectors encoding GFP or GFP-PHB2 fragment (150–206) for 24 h and then analyzed by immunoprecipitation with anti-GFP antibodies. The immunoprecipitants were analyzed by Western blotting with anti-RSU1 and anti-GFP antibodies. *B*, overexpression of PHB2 fragment (150–206) disrupts the RSU1–PHB2 interaction. HT1080 cells were transfected with vectors encoding GFP or GFP-PHB2 fragment (150–206) for 24 h and then analyzed by immunoprecipitation with anti-RSU1 antibodies. The immunoprecipitants were analyzed by Western blotting with anti-PHB2 and RSU1 antibodies. Note that the amount of PHB2 coimmunoprecipitated with RSU1 was reduced in the presence of the GFP-PHB2 fragment (150–206) (compare lanes 5 and 6). *C* and *D*, overexpression of the GFP-PHB2 fragment (150–206) (*C*) but not that of the GFP-PHB2 fragment (201–299) (*D*) attenuates cell–ECM detachment–induced suppression of MEK-ERK activation. HT1080 cells transfected with vectors encoding GFP, GFP-PHB2 (150–206), or GFP-PHB2 (201–299) for 24 h were trypsinized and replated on fibronectin (10 μg/ml) (Adh) or maintained in suspension in HEMA-coated cell culture dishes (Sus) for 5 h. The cells were analyzed by Western blotting as indicated. The levels of total MEK and ERK and phosphorylated MEK^Ser 221^ and ERK^Thr202/Tyr204^ were assessed and quantified as in Figure 1. n = 4 to 9 experiments. ∗∗∗∗*p* < 0.0001, ∗∗*p* < 0.005, ∗*p* < 0.01. ECM, extracellular matrix; ERK, extracellular signal–regulated kinase; NS, not significant.

We next sought to test whether RSU1 interaction with PHB2 is involved in cell–ECM detachment–induced suppression of ERK activation. Consistent with a critical role of PHB2 binding in RSU1-mediated suppression of ERK activation, expression of venus-tagged PHB2-binding defective RSU1 mutant (amino acids 209–277) (Fig. 2*F*), unlike that of venus-tagged wild-type RSU-1 (Fig. 1*D*, compare lane 4 with lane 3), in RSU1 KO cells, failed to suppress ERK activation in response to loss of cell–ECM adhesion (Fig. 6, compare lane 4 with lane 3). To further test this, we overexpressed GFP-tagged PHB2 fragment (150–206) that contains a major RSU1-binding site (Fig. 2*E*) and GFP alone as a control, respectively, in HT1080 cells (Fig. 7*A*). The GFP-PHB2 fragment (150–206) and GFP were immunoprecipitated from the cells with an anti-GFP antibody. As expected, abundant RSU1 was coimmunoprecipitated with the GFP-PHB2 fragment (150–206) (Fig. 7*A*, lane 4) but not with GFP (Fig. 7*A*, lane 3), confirming that the PHB2 fragment (150–206) binds RSU1. Next, we assessed the effect of overexpression of GFP-PHB2 fragment (150–206) on the interaction between endogenous RSU1 and PHB2 by coimmunoprecipitation. The results showed that overexpression of the GFP-PHB2 fragment (150–206) reduced, although did not eliminate, the interaction between endogenous RSU1 with PHB2 (Fig. 7*B*, compare lanes 5 and 6). Importantly, overexpression of the GFP-PHB2 fragment (150–206) significantly suppressed cell–ECM detachment–induced downregulation of ERK activation (Fig. 7*C*). By contrast, overexpression of the GFP-PHB2 fragment (201–299), which does not bind RSU1 (Fig. 2*E*), did not suppress cell–ECM detachment–induced downregulation of ERK activation (Fig. 7*D*). These results suggest that the interaction of RSU1 with PHB2 is critical for cell–ECM detachment–induced downregulation of ERK activation. However, consistent with the fact that overexpression of the GFP-PHB2 fragment (150–206) reduced but did not eliminate the interaction between endogenous RSU1 with PHB2 (Fig. 7*B*, lanes 5 and 6), overexpression of the GFP-PHB2 fragment (150–206), unlike loss of RSU1 (Fig. 1*C*), did not completely eliminate the cell–ECM detachment–induced downregulation of ERK activation (Fig. 7*B*, lower panel). The cell–ECM detachment–induced downregulation of MEK activation was also alleviated in response to overexpression of the GFP-PHB2 fragment (150–206) (Fig. 7*C*, lanes 2 and 4), although the effect was smaller than that induced by the loss of RSU1 (Fig. 1*C*).

**Figure 8:**
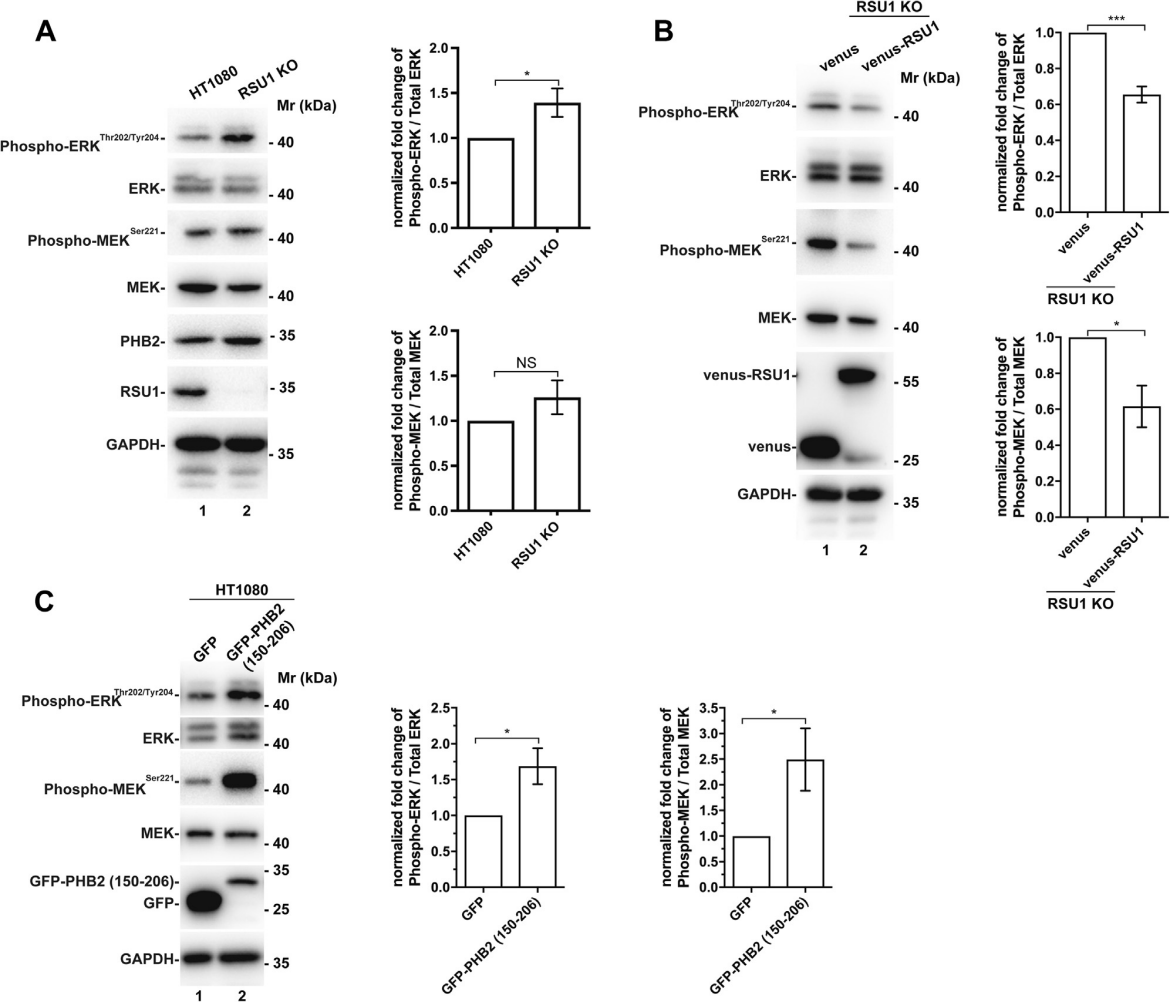
**RSU1 and its interaction with PHB2 interaction suppresses ERK activation in cells grown in 3D culture.***A*, wild-type or RSU1 KO HT1080 cells were grown in 3D culture as described in Experimental procedures. The levels of total MEK and ERK and phosphorylated MEK^Ser 221^ and ERK^Thr202/Tyr204^ were determined by Western Blotting. The densiometric ratio of phosphorylated MEK^Ser 221^ to the total MEK and that of phosphorylated ERK^Thr202/Tyr204^ to the total ERK were analyzed as in Figure 1. n = 3 experiments, ∗*p* < 0.05. *B*, re-expression of RSU1 in RSU1 KO cells suppresses ERK activation in cells grown in 3D culture. RSU1 KO cells stably expressing venus-RSU1 or venus alone were cultured in 3D, and the levels of total MEK and ERK and phosphorylated MEK^Ser 221^ and ERK^Thr202/Tyr204^ were assessed as in (*A*). n = 4 experiments, ∗∗∗*p* < 0.001, ∗*p* < 0.05. *C*, overexpression of the GFP-PHB2 fragment (150–206) that reduces the RSU1–PHB2 interaction increases ERK activation in cells grown in 3D culture. HT1080 cells transfected with GFP or GFP-PHB2 (150–206) for 24 h were trypsinized and then cultured in 3D as described in Experimental procedures. The levels of total MEK and ERK and phosphorylated MEK^Ser 221^ and ERK^Thr202/Tyr204^ were assessed and quantified as in (*A*). n = 6 experiments. ∗∗∗*p* < 0.001, ∗*p* < 0.05. ECM, extracellular matrix; ERK, extracellular signal–regulated kinase; KO, knockout; NS, not significant.

Additionally, we have analyzed the role of RSU1 and its interaction with PHB2 in regulation of ERK activation in cells grown in 3-dimentional (3D) culture. The results show that RSU1 KO cells grown in 3D culture exhibited significantly increased ERK activation compared with wild-type cells that express RSU1 (Fig. 8*A*, compare lane 2 with lane 1). Furthermore, re-expression of venus-tagged RSU1 but not venus alone in RSU1 KO cells grown in 3D culture suppressed ERK activation (Fig. 8*B*, compare lane 2 with lane 1). Finally, overexpression of dominant negative GFP-PHB2 fragment (150–206), which inhibits RSU1 interaction with PHB2 (Fig. 7*B*), in cells grown in 3D culture markedly increased ERK activation (Fig. 8*C*, compare lane 2 with lane 1). These results suggest that RSU1, through its interaction with PHB2, suppresses ERK activation in cells grown in 3D culture.

### RSU1 is a multifunctional protein that is positively involved in cell spreading, directional migration, and invasion

**Figure 9:**
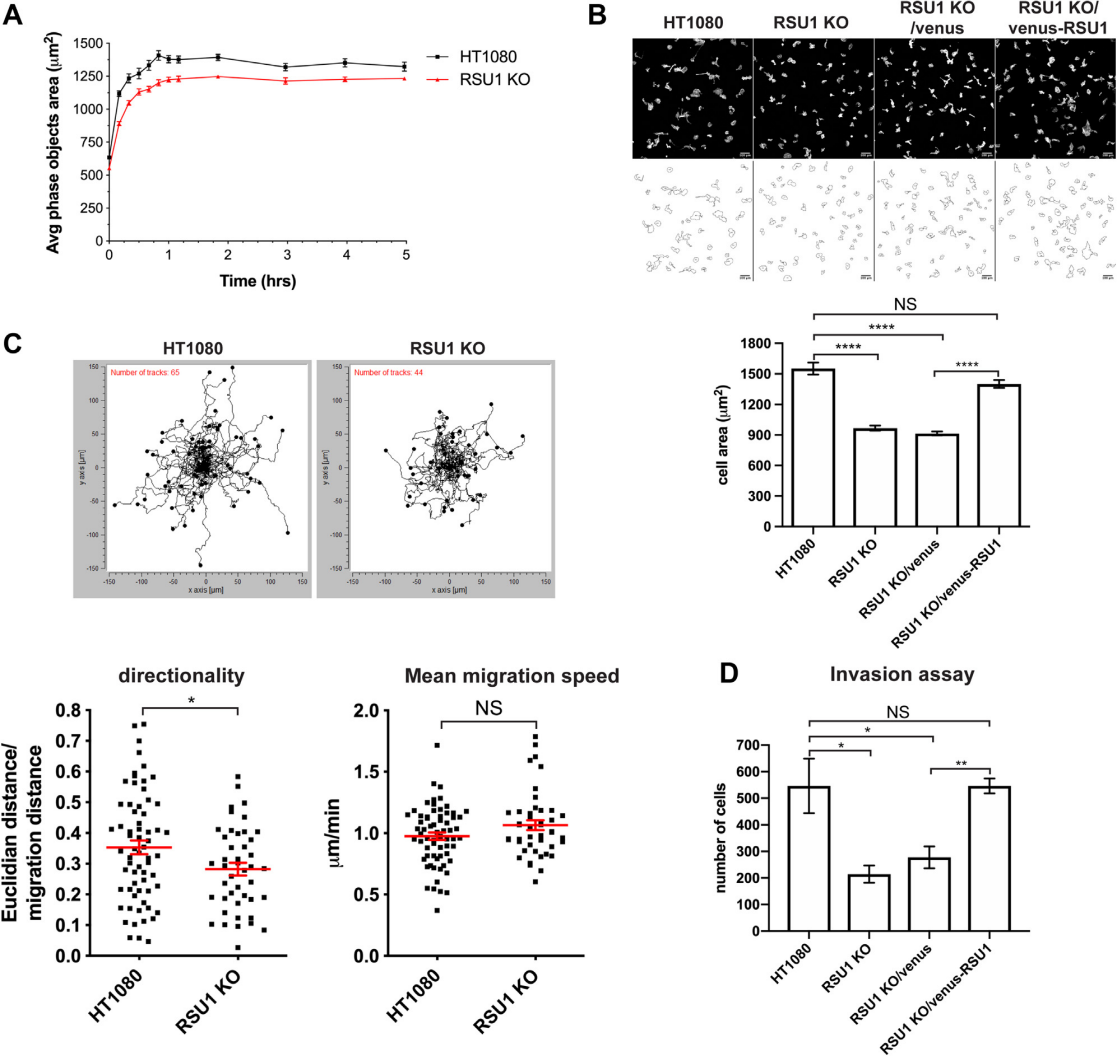
**RSU1 promotes cell spreading, directional migration, and invasion.***A*, the spreading of wild-type and RSU1 KO HT1080 cells on culture dishes coated with fibronectin (30 μg/ml) were analyzed with IncuCyte ZOOM apparatus over the course of 5 h. Cell surface areas (mean ± SE) of wild-type HT1080 cells and RSU1 KO cells were quantified as described in Experimental procedures. *B*, cells (as indicated in the figure) were seeded on fibronectin (30 μg/ml)-coated culture dishes overnight and then stained with Alexa 32 Fluor555–conjugated phalloidin. Cell area was quantified using ImageJ software (NIH). Data are shown as mean ± SEM of cell areas measured in 4 to 6 randomly selected fields. ∗∗∗∗*p* < 0.0001. *C*, cell tracks of migrating wild-type HT1080 (n = 65) and RSU1 KO (n = 44) cells. The directional persistence (directionality) and mean migration speed during cell migration were measured. The mean ± SEM is plotted in *red*. ∗*p* < 0.05. *D*, wild-type HT1080, RSU1 KO, and RSU1 KO cells expressing venus or venus-RSU1 were analyzed in an invasion assay as described in Experimental procedures. ∗*p* < 0.05, ∗∗*p* < 0.01. KO, knockout; NS, not significant.

Upon cell–ECM adhesion, RSU1 is known to localize to cell–ECM contact sites, which are critical for cell spreading, migration, and invasion. Thus, we have analyzed the effects of loss of RSU1 on these processes using the RSU1 KO cells. The spreading of wild-type and RSU1 KO HT1080 cells was analyzed using time-lapse live-cell imaging. Consistent with previous studies (56, 57), loss of RSU1 reduced cell spreading on fibronectin (Fig. 9*A*). Re-expression of venus-RSU-1 but not venus in RSU-1 KO cells effectively restored cell spreading (Fig. 9*B*). These results suggest that RSU-1 is positively involved in cell spreading.

Next, we assessed the effect of RSU1 deficiency on cell migration by live-cell imaging. The migration speed and directionality (*i.e.*, directional persistence, which can be determined by the ratio of Euclidian distance to accumulated migration distance for each track (58)) of individual cells were tracked. The results showed that while the mean migration speed was not reduced in RSU1 KO cells compared with that of wild-type HT1080 cells, the directional persistence was significantly impaired in response to loss of RSU1 (Fig. 9*C*). Consistent with the impaired directionality of cell migration, the invasion of RSU1 KO cells through matrigel was inhibited in response to loss of RSU1, which was restored when venus-tagged RSU1 was re-expressed in RSU1 KO cells (Fig. 9*D*). Taken all together, these results suggest that RSU1 is positively involved in cancer cell spreading, directional migration, and invasion through ECM.

## Discussion

Aberrant activation of ERK signaling, which is critical for control of cell behavior including cell growth, migration, and survival, plays a pivotal role in the pathogenesis and progression of cancer (9, 59, 60). It has been well established that cell–ECM adhesion exerts strong influence on ERK activation and detachment of cells from the ECM often results in downregulation of ERK activation (1, 2, 3, 4, 5, 6, 7, 8, 9). Indeed, aberrant ERK signaling contributes to anchorage-independent cell growth, a hallmark of malignant transformation. However, despite the importance, our understanding of the molecular mechanism by which cell–ECM detachment regulates ERK activation remains rather incomplete. The studies presented in this paper have shed important new insights into this process. Specifically, we have identified RSU1, an evolutionally conserved protein localized to cell–ECM adhesion, as a key mediator of cell–ECM detachment–induced downregulation of ERK activation. KO of RSU1 abolished cell–ECM detachment–induced downregulation of ERK activation. Thus, cells lacking RSU1 exhibited a constitutively high level of ERK activation despite the absence of cell–ECM adhesion (Fig. 1). Additionally, loss of RSU1 results in increases of ERK activation in cells cultured in 3D (Fig. 8). Together, these findings suggest a suppressive role of RSU1 in ERK activation, which provides an explanation as to why loss or reduced expression or mutations of *RSU1* was often found in human cancers (*e.g.*, hepatocellular carcinoma and gliomas) (37, 38, 39) and why overexpression of RSU1 can inhibit anchorage-independent growth of human cancer cells (39, 40).

**Figure 10:**
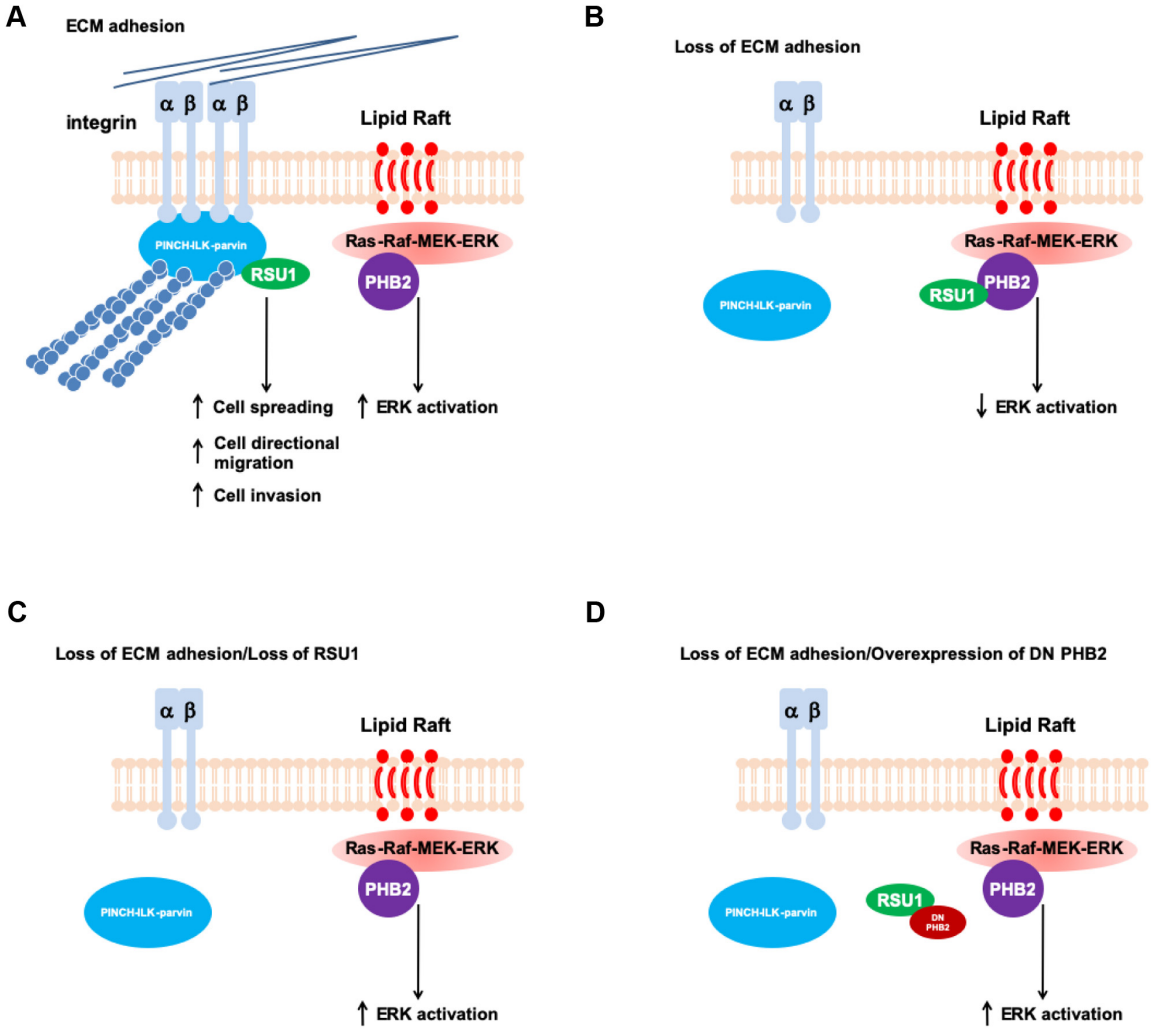
**A model of RSU1-mediated regulation of ERK signaling in response to changes of cell–ECM adhesion.** The figure depicts a model in which RSU1 regulates ERK signaling in response to changes of cell–ECM adhesion. In cells with abundant cell–ECM adhesions, RSU1 is concentrated in cell–ECM adhesions where it interacts with the ILK–PINCH–parvin complex and facilitates cell spreading, directional migration, and invasion (*A*). Loss or reduction of cell–ECM adhesion releases RSU1 from cell–ECM adhesions, resulting in increased RSU1 association with membrane lipid rafts and interaction with PHB2 and consequently suppression of ERK activation (*B*). This negative regulatory mechanism is compromised in cells lacking RSU1 (*C*) or overexpressing dominant negative PHB2 fragment (DN) that inhibits the RSU1–PHB2 interaction (*D*), resulting in aberrant activation of ERK despite the absence or reduction of cell–ECM adhesion. ECM, extracellular matrix; ERK, extracellular signal–regulated kinase; PINCH, particularly interesting new cysteine-histidine-rich protein.

How does RSU1 mediate cell–ECM detachment–induced downregulation of ERK signaling? The findings presented in this paper suggest that RSU1 functions in this process through PHB2. PHB2 and its homologous protein PHB1 are assembled into a large hetero-oligomeric PHB protein complex localized in various subcellular locations including membrane lipid rafts (49, 50). Substantial evidence indicates that the PHB complex is involved in multiple biological processes that impact tumorigenesis (50, 61). Of note, previous studies have shown that the PHB complex is required for Ras-induced MEK and ERK activation (51, 53). Indeed, consistent with the previous studies (51, 53), knockdown of PHB2 reduced MEK and ERK activation (Fig. 3*A*). Moreover, depletion of PHB2 uncoupled MEK and ERK activation from cell–ECM adhesion (Fig. 3*B*), underscoring the importance of PHB2 in this process. We have found that RSU1 directly binds to PHB2 (Fig. 2). Furthermore, the interaction of RSU1 with PHB2 was significantly increased in response to cell–ECM detachment. Expression of PHB2-binding defective RSU1 mutant (209–277) (Fig. 6), unlike that of wild-type RSU1 (Fig. 1*D*), in RSU1 KO cells, failed to restore cell–ECM detachment–induced downregulation of ERK signaling. Inhibition of RSU1 interaction with PHB2 by ectopic expression of a dominant negative PHB2 fragment that reduces the RSU1–PHB2 interaction also alleviated cell–ECM detachment–induced downregulation ERK activation (Fig. 7). Consistent with this, overexpression of the dominant negative PHB2 fragment increased ERK activation in cells grown in 3D (Fig. 8). Collectively, our findings suggest a model in which the interaction of RSU1 with PHB2 serves as a sensor for the status of cell–ECM adhesion and links it to the regulation of ERK signaling (Fig. 10). In this model, in cells with abundant cell–ECM adhesions, RSU1 is concentrated in cell–ECM adhesions where it interacts with the ILK–PINCH–parvin complexes and promotes cell spreading, directional migration, and invasion (Fig. 10*A*). Loss or reduction of cell–ECM adhesion releases RSU1 from cell–ECM adhesions, resulting in increased RSU1 association with membrane lipid rafts and interaction with PHB2 and consequently suppression of ERK activation (Fig. 10*B*). This negative regulatory mechanism is compromised in cells lacking RSU1 (Fig. 10*C*) or overexpressing dominant negative PHB2 fragment that inhibits RSU1–PHB2 interaction (Fig. 10*D*), resulting in aberrant activation of ERK despite the absence or reduction of cell–ECM adhesion. This model may provide an explanation for the initial observation, based upon which the name Ras Suppressor 1 (RSU1) was coined, that overexpression of RSU1 suppresses *v*-Ki-Ras–induced oncogenic transformation (29) as well as clinical observations that loss or reduced expression or mutations of *RSU1* was associated with certain types of human cancers (*e.g.*, hepatocellular carcinoma and gliomas) (37, 38, 39). While this model is supported by both the biochemical and FRET analyses of the RSU1–PHB2 interaction and the functional studies with three different experimental approaches (*i.e.*, RSU1 KO, PHB2 knockdown and overexpression of the GFP-PHB2 fragment (150–206) that reduces the RSU1–PHB2 interaction), our studies do not exclude the possibility that other RSU1-mediated interactions might also be involved in this process. In this regard, it is worth noting that while loss of RSU1 completely eliminated the effect of cell–ECM detachment on ERK activation (Fig. 1*C*), overexpression of the GFP-PHB2 fragment (150–206) only partially suppressed the effect of cell–ECM detachment on ERK activation (Fig. 7*C*). While this partial reversion could be due to technical reasons (*e.g.*, incomplete inhibition of the RSU1–PHB2 interaction by the GFP-PHB2 fragment (150–206) (Fig. 7*B*), it is possible that other RSU1-mediated interactions might also contribute to this process.

Our studies have demonstrated that the interaction of RSU1 with PHB2 is influenced to a great extent by cell–ECM adhesion (Fig. 5). How does the interaction of RSU1 with PHB2 sense the status of cell–ECM adhesion? Previous studies have shown that cell–ECM adhesion exerts a strong effect on membrane order through a process that depends on both membrane lipid rafts and clustering of focal adhesion proteins (62). PHB2 is a component of membrane lipid rafts (49, 50). Furthermore, it has been shown that membrane lipid rafts are involved in cell–ECM detachment–induced downregulation of ERK activation (47, 63). In the studies presented here, we showed that a fraction of RSU1 was associated with membrane lipid rafts. Importantly, although the amount of PHB2 in membrane lipid rafts was not significantly altered in response to cell–ECM detachment, the amount of RSU1 associated with membrane lipid rafts was increased in this process (Fig. 4). Concomitantly, the interaction of RSU1 with PHB2 was enhanced (Fig. 5). Thus, it is attractive to propose that the interaction of RSU1 with PHB2 senses the status of cell–ECM adhesion through, at least in part, alteration of membrane lipid rafts. Clearly, future studies are required to further test this possibility.

Given the central role of aberrant ERK signaling in the development and progression of cancer (9, 59, 60), our findings that RSU1, whose expression is frequently lost or reduced in human cancers (37, 38, 39), binds PHB2 and consequently mediates cell–ECM detachment–induced suppression of ERK signaling have important implications for development of therapeutic agents that control cancer progression. Indeed, recent studies have shown that rocaglamides, a class of natural anticancer compounds, directly bind PHB1 and PHB2 and inhibit ERK activation (54). Thus, the RSU1-PHB signaling axis identified in the current study may serve as an effective target for therapeutic control of aberrant ERK signaling, anchorage-independent growth, and cancer progression.

## Experimental procedures

### Cell culture

Human HT1080 fibrosarcoma cells were from ATCC and cultured in cell culture dishes with minimum essential medium supplemented with 10% fetal bovine serum (FBS), 2 mM L-glutamine, 0.1 mM nonessential amino acids, 1.0 mM sodium pyruvate, and penicillin and streptomycin with 50 U/ml each at 37 °C in 5% CO_2_ and 3% O_2_. Transfection was performed using LipofectAMINE 3000 (Invitrogen) according to the manufacturer's instructions. For cells cultured in adhesion and suspension, respectively, trypsinized cells were either allowed to adhere to fibronectin (10 μg/ml) or maintained in suspension in hydroxyethyl methacrylate-coated (12 mg/ml, Sigma) non-adhesive cell culture dishes for 5 h. For 3D cell culture, type I collagen (corning 354249) was diluted with sterile deionized water to a final concentration of 3.0 mg/ml. Cells (as specified in each experiment) were harvested with trypsin, washed, and mixed with collagen I (the final cell density = 5.0 × 10^5^ cells/ml and the final concentration of collagen I = 1.0 mg/ml), and pH was adjusted to 7.4 with 1 M NaOH. The cells were cultured in 3D collagen I gels for 24 h and then transferred into tubes and treated with 1 mg/ml collagenase D (Roche, 37334226) for 0.5 h at 37 °C. The cells were collected by centrifugation at 300*g* for 3 min and lysed with the radio-immunoprecipitation assay buffer for further analyses.

### Antibodies, siRNAs, and other reagents

Rabbit polyclonal anti-RSU1 antibody used for immunoprecipitation was from BETHYL (Montgomery, AL). Mouse monoclonal anti-PINCH-1 antibody was from BD. Mouse monoclonal anti-GAPDH antibody was from Abmart (Berkeley, NJ). Rabbit monoclonal anti-MEK, anti-phosphorylated MEK^Ser 221^, anti-ERK, anti-phosphorylated ERK^Thr202/Tyr204^, anti-caveolin-1, and anti-PHB2 antibodies used for Western blotting were purchased from Cell Signaling. Alexa fluor647-conjugated goat anti-mouse IgG antibody was from ThemoFisher Scientific. Horseradish peroxidase–conjugated secondary antibodies were from Jackson Immuno Research Laboratories (West Grove, PA). Alexa555-CTxB used to label lipid rafts was purchased from Invitrogen (Carlsbad, CA).

Small interfering RNA (siRNA) specifically targeting RSU1 and PHB2, respectively, and their corresponding scramble control siRNA were purchased from Genepharma (Shanghai). The sequences of synthetic siRNAs directed against PHB2 are siPHB2-1: 5′-gccucaucaaggguaagaatt-3′ and siPHB2-2: 5′-gugauuuccuacaguguuguucccu-3′, respectively. siRNA was transfected using RNAiMax (Invitrogen) following the manufacturer's protocol. Experiments were carried out 48 h after the transfection.

Restriction endonucleases were obtained from New England Biolabs, Inc (Beverly, MA). Cell culture media and corresponding items were from Sigma-Aldrich (St Louis, MO) or Invitrogen (Carlsbad, CA). All other chemicals were from Fisher Scientific (Fairlawn, NJ) or Sigma-Aldrich (St Louis, MO).

### Generation of RSU1 KO cell lines

HT1080 cells in which *RSU1* was knocked out were generated by CRISPR/cas9-mediated gene disruption. Briefly, a guide RNA (gRNA) oligo designed to target the sequence of 5′-ccggggcatctccaacatgctgg-3′ located at the exon 1 of *RSU1* was cloned into pSpCas9 (BB) -2a-GFP (PX458 containing *cas9*, was a gift from Dr Feng Zhang, Addgene plasmid # 48138) via *Bbs*I sites and transfected into HT1080 cells. Single GFP-positive cells were sorted into each wells of 96-well plates by FACS sorter (BD FACS Aria III). Once the single colonies are propagated, PCR-based analyses as well as Western blotting were employed to assess targeted gene disruption of *RSU1*. Genomic DNA of individual colonies was extracted and amplified with a pair of DNA oligos flanking the gRNA-targeting site, *i.e.*, P1: 5′-ccaaccctggggaagcctcaga-3′ and P2: 5′-tactgcaaaccctctgcgcg-3′ for RSU1. The PCR products were subcloned into pTA vectors. For each single colony, 6 to 8 clones were selected for DNA sequencing, and Western blotting using anti-RSU1 antibody was used to further verify the RSU1 KO cell line.

### Generation of RSU1-Clover knock-in cell lines

The DNA sequence encoding Clover was inserted into exon 8 of *RSU1* in HT1080 cells using CRISPR/cas9-mediated targeted gene editing and homology-mediated recombination. Briefly, a guide RNA (gRNA) oligo (5′-caccgaaagatcagccggaaacccc-3′) designed to target the sequence (5′-aaagatcagccggaaacccctgg-3′) located at the exon 8 of *RSU1* was cloned into pSpCas9 (BB) -2a-GFP (PX458 containing *cas9*, Addgene plasmid # 48138) via *Bbs*I sites. The engineered pUC19 vector carried Clover sequence–flanked respectively, by the 600 to 700 bp of DNA sequences immediately upstream and downstream of the CRISPR/cas9 targeting site was served as donor for homology-mediated recombination. The guide RNA targeting site (*i.e.*, 5′-aaagatcagccggaaacccctgg-3′) was also inserted to the 5′ and 3′ of the homolog sequences, respectively, to allow the linearization of the vector by Cas9 and the same guide RNA. Single GFP-positive cells were sorted into each well of 96-well plates by FACS sorter (BD FACS Aria III). Once the single colonies are propagated, PCR-based analyses as well as Western blotting were employed to assess targeted gene knock-in.

### Generation of mouse monoclonal anti-RSU1 antibody

Mouse monoclonal antibodies recognizing RSU1 were prepared using GST-fusion proteins containing RSU1 residues 1 to 299 (full-length) as an antigen based on a previously described method (24, 65). Hybridoma supernatants were initially screened for anti-RSU1 activities by ELISA using MBP-RSU1 protein. Positive clones were selected and further tested by Western blotting using GFP- and FLAG-tagged RSU1.

### DNA cloning

The ORFs of RSU1 and PHB2, respectively, were amplified by PCR and subloned into mClover-N1 (Addgene, no. 54538) and mRuby2-N1 (Addgene, no. 54614). DNA fragments encoding PHB2 deletion mutants were generated by PCR and were cloned into pEGFP-C1. All DNA constructs were confirmed by DNA sequencing.

### Yeast two-hybrid screen

A full-length RSU1 cDNA was cloned from a human Kidney cDNA library (Clontech) by PCR using the following primers: 5ʹ-gcgaattcatgtccaagtctctgaagaagttggtg-3ʹ and 5ʹ-cggtcgacttatctgttcttggctgccaggggtttcc-3ʹ. The sequence of the RSU1 cDNA was confirmed by automated DNA sequencing. The RSU1 cDNA was inserted into the pGBKT7 vector (Clontech). The pGBKT7/RSU1 construct was used as bait to screen a human keratinocyte MATCHMAKER cDNA library following a previously described protocol (24, 64, 65). Six positive plasmids containing cDNA inserts with an identical size (1.1 kb) were selected and sequenced. They contain an identical cDNA fragment encoding the C-terminal fragment of PHB2 (residues 101–299). The full-length PHB2 cDNA was isolated from the human lung cDNA library by PCR using the following primers: 5ʹ-gctgaattcatggcccagaacttgaaggacttgg-3ʹ and 5ʹ-gagctcgagtcatttcttacccttgatgaggctg-3ʹ.

### Lipid raft isolation

Lipid rafts were isolated from HT1080 fibrosarcoma cells using MinuteTM Lipid Raft Isolation Kit for Mammalian Cells/Tissues (LR-039, Invent Biotechnologies, MN) following the manufacturer's instructions. Briefly, 30 × 10^6^ cells were collected and spun down at 500*g* for 5 min. The cell pellet was resuspended in 500 μl of buffer A and vortexed for 20 s. The cell suspension was centrifuged in the filter cartridge at 16,000*g* for 30 s. The pellet was then resuspended and centrifuged at 1000*g* for 5 min. The pellet containing nuclei, large cell debris, and some unruptured cells was discarded, and the supernatant was centrifuged further at 16,000*g* for 30 min. The resulting supernatant was saved as the cytosolic fraction, and the total membrane fraction in the pellet was resuspended in buffer B and incubated on ice for 30 min before centrifugation at 16,000g for 10 min. The supernatant was collected and mixed with buffer C to be subjected to centrifugation at 10,000*g* for 10 min, resulting in lipid rafts floating on the top of the tube. The aqueous phase was removed, and the lipid rafts was resuspended in buffer A and was centrifuged at 16,000*g* for 5 min. Finally, the lipid rafts in the pellet were resuspended with the radio-immunoprecipitation assay buffer (10 mM Tris-HCl pH 8.0, 0.5 mM EGTA, 1 mM EDTA pH 8.0, 1% TritonX-100, 0.1% sodium deoxycholate, 0.1% SDS, 140 mM NaCl).

### Lipid raft staining

Lipid rafts were labeled by Vybrant Alexa Fluor 555 Lipid Raft Labeling Kits (Invitrogen, CA) following the manufacturer's instructions. In brief, cells were incubated with fluorescent CTxB conjugate working solution after washing with complete growth medium at 4 °C. After the incubation, cells were spun down and then were resuspended with prechilled anti-CTxB antibody working solution at 4 °C for 15 min. Cells were fixed with ice-cold PBS containing 4% formaldehyde, and lipid rafts were visualized by fluorescence microscopy.

### Generation of GST- or MBP-tagged RSU1 and PHB2 fusion proteins

The ORF of RSU1 and PHB2 was inserted into the pGEX-5x-1 vector (Pharmacia), respectively. The full-length or deletion mutant forms of PHB2 were inserted into the pMAL-C2 vector (New England BioLabs), respectively. The recombinant vectors were used to transform *E. coli* cells. The expression of the GST- or MBP-fusion proteins was induced with IPTG. GST- and MBP-tagged fusion proteins were isolated using glutathione-Sepharose 4B and amylose-agarose beads, respectively, as we previously described (24, 65).

### GST-fusion protein binding assays

The association of PHB2 with endogenous RSU1 derived from mammalian cells was analyzed using a GST–PHB2 fusion protein in the pull-down assay as we previously described (65). Briefly, HeLa cells from three100-mm plates were lysed with 6 ml of lysis buffer (1% TX-100 in 50 mM Hepes (pH 7.1) containing 150 mM NaCl, 10 mM Na_4_P_2_O_7_, 2 mM Na_3_VO_4_, 100 mM NaF, 10 mM EDTA, and protease inhibitors (final concentrations of 1 μg/ml aprotinin, 10 μM leupeptin, 1 μM pepstatin, and 1 mM PMSF). The lysates were precleared with glutathione-Sepharose 4B beads. The precleared lysates (1.4 mg total amount of protein) were mixed with glutathione-Sepharose 4B beads containing GST-PHB2 or GST (25 μl) and incubated at 4 °C overnight. After washing, proteins coprecipitated with GST-PHB2 were detected by Western blotting with anti-RSU1 antibody.

To analyze direct interaction between RSU1 and various mutant forms of PHB2, GST-RSU1 (10 μg/ml) were incubated with 25 μl amylose-Sepharose 4B beads containing MBP-tagged full-length or deletion mutant forms of PHB2, or MBP as a negative control in binding buffer (1% Triton X-100, 10 mM Tris, and 100 mM NaH_2_PO_4_, pH 8.0). The mixtures were incubated at 4 °C for 3 h. At the end of incubation, the beads were washed six times with the binding buffer. GST-RSU1 bound to MBP-tagged full-length or deletion mutant forms of PHB2 was detected by Western blotting with an anti-RSU1 antibody.

### Western blotting

Western blotting was performed as previously described (64). In brief, whole-cell proteins were extracted using the radio-immunoprecipitation assay buffer (10 mM Tris-HCl pH 8.0, 0.5 mM EGTA, 1 mM EDTA, 1% Triton X-100, 0.1% sodium deoxycholate, 0.1% SDS, 140 mM NaCl). The concentration of total proteins was determined using BCA protein assay kit (Pierce). Ten to 30 μg of proteins was loaded per lane. The proteins were separated by SDS-PAGE and transferred to a polyvinylidene fluoride membrane (Millipore). The membranes were blocked with 5% BSA in Tris-buffered saline (50 mM Tris-HCl and 150 mM NaCl, pH 7.4) containing 0.1% Tween 20 for 1 h at room temperature, followed by overnight incubation at 4 °C with a specific primary antibody. After washing and incubating with appropriate horseradish peroxidase–conjugated secondary antibodies (anti-mouse, 711-005-152, or anti-rabbit, 715-005-151; Jackson ImmunoResearch), blots were developed using an ECL kit (Bio-Rad) and then exposed to an x-ray film (super RX-N-C, Fuji Film).

### Coimmunoprecipitation assay

Cells were lysed with the lysis buffer containing 10 mM Tris/HCl, pH 7.6, 500 mM NaCl, 1.75% n-octyl-β-D-glucopyranoside, and protease inhibitors cocktails (Roche) as specified. The cell lysates were mixed with agarose beads conjugated with anti-RSU1 (4 μl) or anti-PHB2 antibody (5 μl). The beads were washed four times, and the immunoprecipitates were analyzed by Western blotting with antibodies as specified.

### Immunofluorescent staining

Human HT1080 cells were fixed with 4% paraformaldehyde in PBS, permeabilized with 0.1% Triton X-100 in PBS, and stained with the primary rabbit monoclonal anti-PHB antibody and Alexa fluor647–conjugated anti-mouse IgG antibody. The cells were observed under a Nikon A1R confocal microscope equipped with a 100× oil objective.

### Time-correlated single-photon fluorescence lifetime microscopy

Time-correlated single-photon fluorescence lifetime microscopy (TCSPC-FLIM) experiments were performed as follows. HT1080 cells were transiently transfected with mClover-N1-RSU1 and mRuby-N1-PHB2, respectively, and incubated in minimum essential medium in the absence of FBS for 24 h. The cells were fixed with 4% polyformaldehyde, and fluorescence images were recorded with Nikon A1R confocal microscope equipped with a 485-nm pulsed diode laser (PDL 800-D, PicoQuant), a photodetector (PMA Hybrid 40, PicoQuant), and a 100× oil objective. The detection covered a time window of 44 ns after the excitation pulse with the TCSPC resolution of 25.0 ps.

For each group of cells, 33 to 46 cells were analyzed for each experiment. Data analysis was performed by the software "SymPhoTime 64" (PicoQuant). In brief, "n-Exponential Tailfit" was selected as the fitting model, the signals on focal adhesions was selected as ROIs, and "calculated IRF" was selected as initial fit to get the lifetime value (τ_amp_). FRET efficiency (E) was calculated according to the equation E = 1 − τ_DA_/τ_D_, where τ_D_ is the fluorescence lifetime of mClover-N1-RSU1 in the presence of mRuby2-N1-PHB2 and τ_DA_ is the fluorescence lifetime of mClover-N1-RSU1 in the presence of mRuby2-N1.

### Quantification of Western blotting and statistical analysis

The chemiluminescent blots were imaged with the Tanon 6100B imager (Tanon, Shanghai, China). ImageJ software (NIH) was used to select and determine the density of the bands showing total levels of MEK and ERK and phosphorylated MEK (Ser 221) and ERK (Thr202/Tyr204) in all the blots. The densiometric ratio of phosphorylated MEK to total MEK and that of phosphorylated ERK to total ERK were calculated and normalized to the control in each experiment. Statistical analysis of the quantification data was expressed as means ± SEM. Differences between the groups were examined for statistical significance using one- or two-tailed paired *t* test (GraphPad Prism, version 5.00, for Windows, GraphPad Software, La Jolla, CA). A value of *p* < 0.05 was considered to be statistically significant.

### Cell spreading assay using IncuCyte ZOOM apparatus

To monitor cell spreading, 2 × 10^4^ cells were seeded in 6-well plates that were precoated with fibronectin (30 μg/ml) and placed into IncuCyte ZOOM apparatus (Essen BioScience, MI). Images of the collective cell spreading at 16 different locations were captured every 15 min for a total duration of 8–10 h using the IncuCyte ZOOM live-cell imaging system (Essen BioScience, MI). After 2 h of plating, the cell surface area does not show significant variation over time. Therefore, the mean value of near-maximal cell surface area was calculated by averaging the values that show no significant variation over the time (up to 5 h after plating).

### Cell spreading area analysis using actin staining

Cell spreading was quantified by measuring the cell area (μm^2^) as described previously (66). A two-tailed paired Student's *t*-test was performed to test for significance in all experiments.

### Cell migration

To analyze cell migration, cells were seeded in a culture dish coated with fibronectin (30 μg/ml) and placed in a heated and air-humidified chamber built in a Nikon inverted microscope TE2000E. Phase-contrast time-lapse imaging of a field containing 7 to 10 cells at a 2-min interval for 3 h was captured on the microscpe with a 10× Ph1 objective, perfect focus system (PFS), and Hamamatsu C-11440-22CU camera controlled by NIS-elements software (Nikon). Image stacks were processed using ImageJ software. Cell motility was tracked by using ImageJ (NIH) with plug-in “manual Tracking” (Fabrice Cordelières, Institut Curie, Orsay, France). Data were analyzed by the Chemotaxis-and-Migration-Tool) (ibidi, http://ibidi.com/xtproducts/en/Software-and-Image-Analysis/Manual-Image-Analysis/Chemotaxis-and-Migration-Tool).

### Cell invasion

Cell invasion assay was performed in Corning biocoat matrigel invasion chambers (Corning, NY) with uncoated porous inserts (pore size: 8 μm) according to the manufacturer's protocol. Briefly, cells were plated at a density of 3 × 10^4^ in each well with minimum essential medium free of FBS, and 700 μl of culture medium containing 10% FBS was added to the bottom of the 24-well plate. Following incubation for 24 h, noninvading cells were removed from the upper surface of the membrane using a cotton-tipped swab. The invading cells were subsequently fixed in 4% formaldehyde for 10 min and stained with Hochest 33342 for 5 min. The stained cells were counted as cells per field using Nikon ECLIPSE Ti at 10× magnification in 5 fields.

## Data availability

All data discussed are contained within the manuscript or the supporting material.

## Conflict of interest

The authors declare that they have no conflicts of interest with the contents of this article.
