# Supplementary material for: RSU-1 interaction with prohibitin-2 links cell–extracellular matrix detachment to downregulation of ERK signaling
Source: J Biol Chem. 2020 Dec 3;296:100109. doi: 10.1074/jbc.RA120.014413 (PMC7948471; doi:10.1074/jbc.RA120.014413)
Supplement: Figures S1 to S3 [file mmc1.pdf]

Supporting Information

Fig. S1

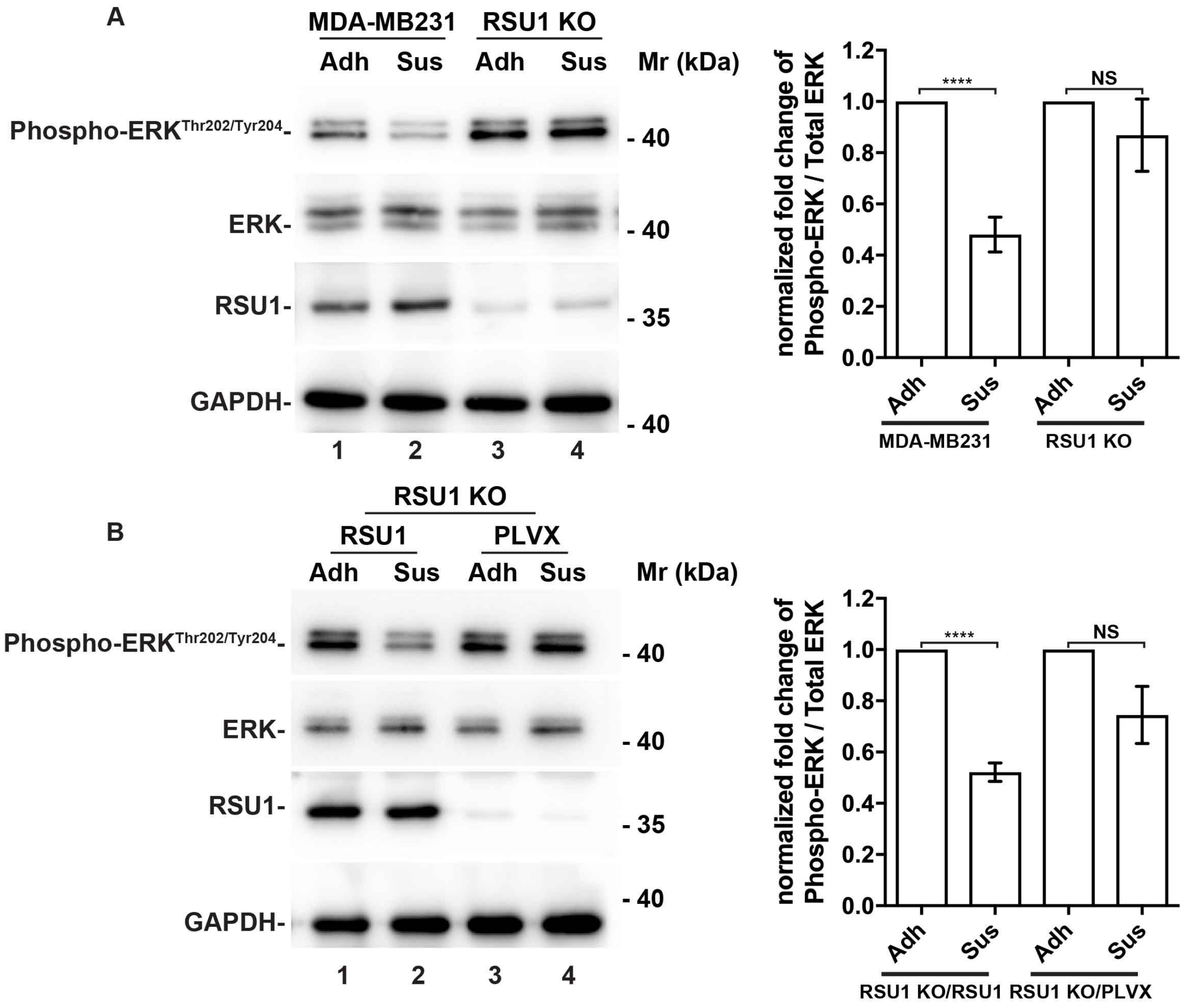

Supplemental Fig. S1. Knockout of RSU1 in MDA-MB231 cells impairs cell-ECM detachment induced downregulation of MEK and ERK signaling

(A) Wild-type and RSU1 KO MDA-MB-231 cells were either allowed to adhere to fibronectin (10 μg/mL) (Adh) or maintained in suspension in HAMA-coated cell culture dishes (Sus) for 5 hrs. The levels of total MEK and ERK, and phosphorylated ERK$^{Thr202/Tyr204}$ were determined by Western Blotting. The densiometric ratio of phosphorylated ERK$^{Thr202/Tyr204}$ to the total ERK were analyzed as described in Materials and Methods. In each data set, data were normalized to that observed in adherent cells. Differences between the attached and suspended cells were analyzed for statistical significance as described in Materials and Methods. n = 5

experiments, **** $p < 0.0001$. (B) Re-expression of RSU1 in RSU1 KO cells restored cell-ECM detachment induced down-regulation of ERK activation. RSU1 KO cells stably transfected with RSU1 expression vector or PLVX empty vector alone were cultured in adhesion and suspension conditions, respectively, and the level of total ERK and phosphorylated $ERK^{Thr202/Tyr204}$ were assessed and quantified as described in (A). n = 4 experiments, **** $p < 0.0001$, NS: not significant.

Fig. S2

RSU1-clover

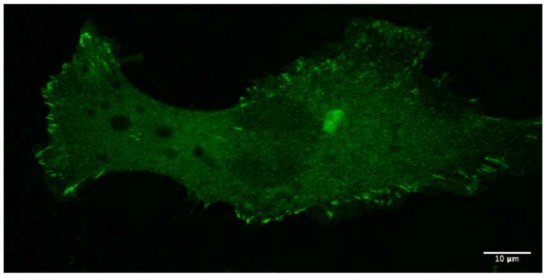

Supplemental Fig. S2. Subcellular localization of RSU1-Clover. RSU1-Clover expressing HT1080 cells, in which the DNA sequence encoding Clover was inserted immediately to the 3'of *RSU1* loci, were generated as described in Materials and Methods. Subcellular localization of RSU1-Clover was analyzed by confocal microscopy.

Fig. S3

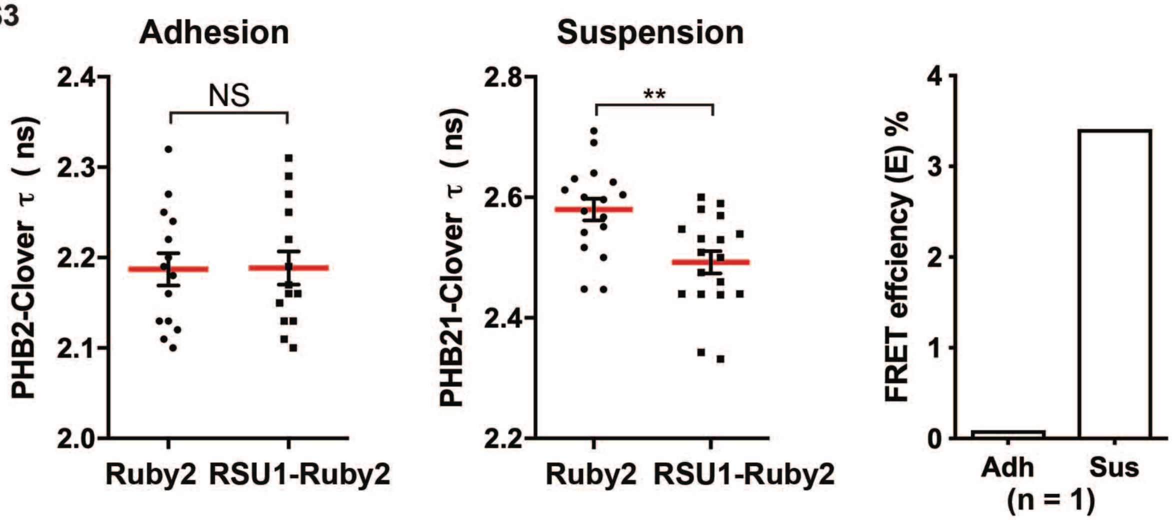

Supplemental Fig. S3. FRET analyses of the RSU1-Ruby2 interaction with PHB2-Clover

HT1080 cells were co-transfected with mClover-N1 vector encoding PHB2 (PHB2-Clover) and Ruby2-N1 vector encoding RSU1 (RSU1-Ruby2) or Ruby2-N1 vector alone as a negative control. The transfected cells were allowed to adhere to fibronectin (10 μg/mL) or

maintained in suspension for 5 hr before fixation with 4% PFA. The fluorescence lifetime (τ) of PHB2-Clover was measured as described in Materials and Methods. The mean ± SEM of τ is plotted. In attached cells, $\tau_{PHB2\text{-}Clover/Ruby2}$ = 2.187 ± 0.018 ns (n = 14), $\tau_{PHB2\text{-}Clover/RSU1\text{-}Ruby2}$ = 2.189 ± 0.018 ns (n = 14). In suspended cells, $\tau_{PHB2\text{-}Clover/Ruby2}$ = 2.58 ± 0.018 ns (n = 17), $\tau_{PHB2\text{-}Clover/RSU1\text{-}Ruby2}$ = 2.492 ± 0.018 ns (n = 18). Noted that in suspended cells, $\tau_{PHB2\text{-}Clover}$ in the presence of RSU1-Ruby2 was significantly reduced compared to that in the presence of Ruby2. ** $p < 0.005$, NS: not significant. FRET efficiency was calculated as described in Materials and Methods. n = 1.
